# Cardiac macrophages prevent sudden death during heart stress

**DOI:** 10.1038/s41467-021-22178-0

**Published:** 2021-03-26

**Authors:** Junichi Sugita, Katsuhito Fujiu, Yukiteru Nakayama, Takumi Matsubara, Jun Matsuda, Tsukasa Oshima, Yuxiang Liu, Yujin Maru, Eriko Hasumi, Toshiya Kojima, Hiroshi Seno, Keisuke Asano, Ayumu Ishijima, Naoki Tomii, Masatoshi Yamazaki, Fujimi Kudo, Ichiro Sakuma, Ryozo Nagai, Ichiro Manabe, Issei Komuro

**Affiliations:** 1 Department of Cardiovascular Medicine, the University of Tokyo, 7-3-1 Hongo, Bunkyo-ku, Tokyo, 113-8655 Japan; 2 Department of Advanced Cardiology, the University of Tokyo, 7-3-1 Hongo, Bunkyo-ku, Tokyo, 113-8655 Japan; 3 PRESTO, Japan Science and Technology Agency, 4-1-8 Honcho Kawaguchi, Saitama, 332-0012 Japan; 4 Medical Device Development and Regulation Research Center, Department of Bioengineering/Department of Precision Engineering, School of Engineering, The University of Tokyo, 7-3-1 Hongo, Bunkyo-ku, Tokyo, 113-8656 Japan; 5 Department of Disease Biology and Molecular Medicine, Graduate School of Medicine, Chiba University, 1-8-1 Inohana, Chuo-ku, Chiba-shi, Chiba, 260-8670 Japan; 6 Jichi Medical University, 3311-1 Yakushiji, Shimotsuke-shi, Tochigi-ken, Tochigi, 329-0498 Japan

**Keywords:** Monocytes and macrophages, Arrhythmias, Heart failure

## Abstract

Cardiac arrhythmias are a primary contributor to sudden cardiac death, a major unmet medical need. Because right ventricular (RV) dysfunction increases the risk for sudden cardiac death, we examined responses to RV stress in mice. Among immune cells accumulated in the RV after pressure overload-induced by pulmonary artery banding, interfering with macrophages caused sudden death from severe arrhythmias. We show that cardiac macrophages crucially maintain cardiac impulse conduction by facilitating myocardial intercellular communication through gap junctions. Amphiregulin (AREG) produced by cardiac macrophages is a key mediator that controls connexin 43 phosphorylation and translocation in cardiomyocytes. Deletion of *Areg* from macrophages led to disorganization of gap junctions and, in turn, lethal arrhythmias during acute stresses, including RV pressure overload and β-adrenergic receptor stimulation. These results suggest that AREG from cardiac resident macrophages is a critical regulator of cardiac impulse conduction and may be a useful therapeutic target for the prevention of sudden death.

## Abstract (web summary)

Cardiac immune cells play various roles in the maintenance of homeostasis and diseases in the heart. Here the authors show that cardiac resident macrophages are a critical regulator of cardiac impulse conduction through amphiregulin production, contributing to the prevention of sudden death.

## Introduction

Heart failure is a growing medical and economic burden worldwide^1,2^. Research and development efforts have focused largely on left ventricular (LV) dysfunction and left heart failure, but right heart failure has been gaining increasing attention as a serious unmet medical need. Right heart failure associates with adverse outcomes in a variety of heart diseases, including left heart failure, in part by compromising cardiac function and worsening serious arrhythmias. Indeed, right ventricular (RV) dysfunction increases the risk for sudden cardiac death^3^. However, the molecular mechanisms for right ventricular (RV) dysfunction remain poorly understood.

Recent studies have revealed the various roles played by immune cells in the maintenance of homeostasis and the development of cardiac diseases. For instance, many reports indicate that macrophages promote left ventricular (LV) remodeling^4^ and, conversely, are required for proper adaptive responses to stress and healing after myocardial infarction^5,6^. Other immune cells such as lymphocytes and mast cells also reportedly play important roles in the LV remodeling^7,8^; however, very little is known about the actions of immune cells during RV stress responses^9^.

Here, we show that cardiac macrophages play an essential role in survival during cardiac stress by maintaining the cardiac electrical conduction through regulating cardiac gap junction formation. These findings may pave the way for a new therapeutic method for sudden death.

## Results

### Macrophages are vital for survival after right heart burden

**Fig. 1:**
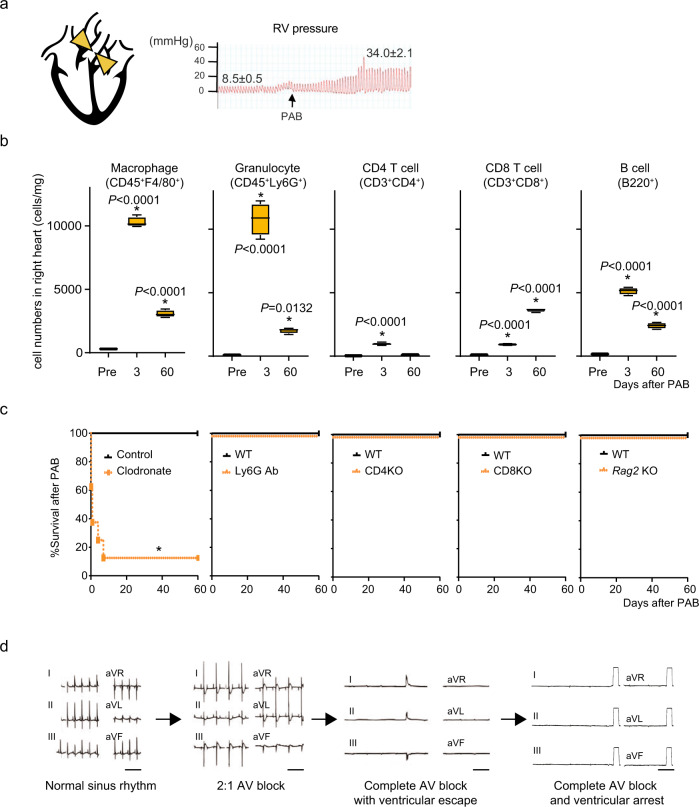
Macrophage depletion causes sudden death after right heart stress. **a** Mouse model of right heart pressure overload (PAB). The pulmonary artery was ligated to increase the RV pressure to 30-35 mmHg from 5-10 mmHg. **b** Flow cytometric analysis of immune cells in the right atrial (RA) and RV free wall in wild-type (WT) mice after PAB. *n* = 4 in each group. One-way ANOVA followed by Tukey’s post-hoc test. *P < 0.05 vs. pre: steady-state. Box plots show center line as median, box limits as upper and lower quartiles, whiskers as minimum to maximum values. **c** Effects of depleting macrophages (clodronate), granulocytes (Ly6G Ab), CD4^+^ (CD4KO) or CD8 T^+^ (CD8KO) cells, or T and B cells (*Rag2*KO) on survival after PAB. *P < 0.0001 (log-rank test). **d** Representative six-channel ECGs from clodronate-treated mice after PAB. Shown is the representative sequence of arrhythmia progression observed in a mouse. Bars, 400 msec.

To assess the functional roles of immune cells during acute RV stress, we imposed pressure overload on the right ventricle by pulmonary artery banding (PAB) (Fig. 1a). Accumulation of various types of immune cells, including macrophages, granulocytes, CD4^+^, and CD8^+^ T cells, and B cells, was observed after PAB (Fig. 1b, Supplementary Fig. 1). To gain insight into the functions of these cell populations during acute RV stress, we performed PAB on mice treated with clodronate liposomes, which depleted or impaired monocytes and macrophages^10^, or with anti-Ly6G antibody, which depleted granulocytes^11^; *Cd4a*^−/−^ and *Cd8a*^−/−^ mice, which lacked CD4^+^ and CD8^+^ T cells, respectively; and *Rag2*^−/−^ mice, which lacked mature T and B cells. While the interventions affecting granulocytes and T and B cells did not impact survival after PAB, more than half (57%) of the clodronate-treated mice died within 4 h after PAB (Fig. 1c). Electrocardiograms (ECGs) showed that, after PAB, 4 of the 7 clodronate-treated mice developed advanced atrioventricular (AV) conduction block, which led to ventricular arrest (Fig. 1d and Supplementary Table 1). To further confirm the involvement of macrophage deficiency in the sudden death phenotype, we used another macrophage depletion model, *Cx*_*3*_*cr1*^*CreER/+*^;*R26*^*iDTR/+*^ mice, in which tamoxifen induces CX_3_CR1^+^ cell-specific expression of the diphtheria toxin receptor^12^. Macrophage depletion in this model also led to sudden death after PAB (Supplementary Figures 2 and 3). Given the sudden death phenotype and advanced AV block seen in clodronate-treated mice, these results strongly suggest that monocytes and macrophages are essential for survival during acute RV pressure overload, presumably by suppressing severe arrhythmias.

### Macrophage-derived AREG protects against sudden death

**Fig. 2:**
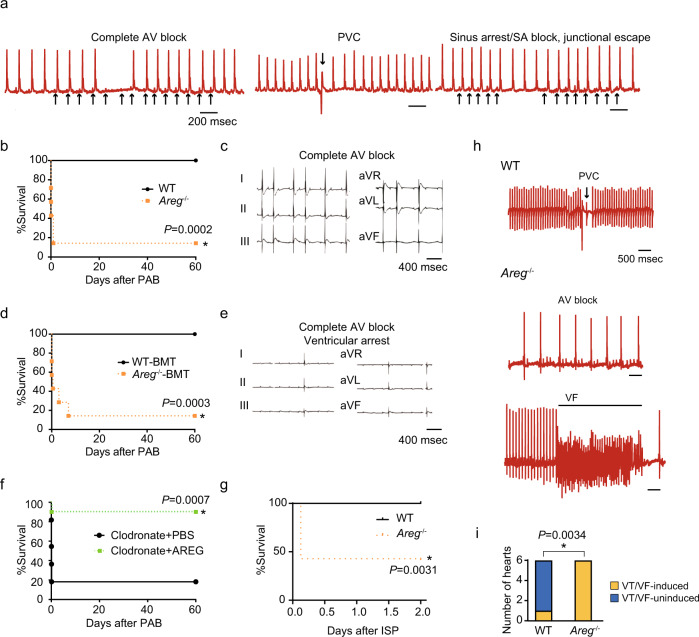
*Areg* deficiency leads to abnormal cardiac electrical conduction at steady-state and lethal arrhythmias under cardiac stress. **a** ECGs from freely moving *Areg*^−/−^ mice recorded using telemetry. Spontaneous AV block, PVCs, and sinus arrest or SA block were observed in *Areg*^−/−^ mice. Bars indicate 200 msec. **b** Survival rates among WT and *Areg*^−/−^ mice after PAB. *P < 0.001, log-rank test. **c** Representative ECG from an *Areg*^−/−^ mouse showing complete AV block and ventricular arrest after PAB. **d**, **e** BM chimeric mice that received WT (WT-BMT) or *Areg*^−/−^ (*Areg*^−/−^-BMT) BM were subjected to PAB. Survival rates after PAB are in **d**. *P < 0.001, log-rank test. In **e**, a representative ECG from an *Areg*^−/−^-BMT mouse after PAB is shown. **f** Survival curves for clodronate-treated mice with or without continuous administration of AREG (5 μg/day) from 24 h before PAB. *n* = 10 in each group. *P < 0.01, log-rank test. **g**, **h** WT and *Areg*^−/−^ mice were intraperitoneally administered isoproterenol (bolus, 5 mg/kg). Survival rates are shown in **g**. In **h**, representative ECGs from WT and *Areg*^−/−^ mice are shown. *P < 0.05, log-rank test. **i** Numbers of Langendorff-perfused hearts that developed VT/VF. *P < 0.01 (χ^2^-test).

We recently demonstrated that cardiac macrophage-derived AREG is essential for the proper cardiac adaptive response to LV pressure overload^5^. Accordingly, we tested whether AREG is similarly important for the response to RV pressure overload. Although we did not observe sudden death in *Areg*^−/−^ mice in the steady-state, continuous ECG monitoring of freely moving 8- to 12-week-old *Areg*^−/−^ mice showed sinus arrest or sinoatrial (SA) block, intermittent complete AV block, and premature ventricular contractions (PVCs) in all 6 mice tested (Fig. 2a and Supplementary Table 2). By contrast, no arrhythmias were observed in wild-type (WT) mice (*n* = 6). When we imposed RV pressure overload on *Areg*^−/−^ mice with PAB, the mice developed AV block, and >85% of them died within 24 h (Fig. 2b, c and Supplementary Table 3) with no noticeable histological or functional changes in their RVs (Supplementary Figs. 4 and 5). To confirm that macrophage-derived AREG is important for preventing arrhythmias, we transplanted bone marrow (BM) from WT or *Areg*^−/−^ mice into lethally irradiated WT mice. Six weeks after the BM transplantation (BMT), 99.2% of the cardiac-resident macrophages were replaced with BM-derived cells. After PAB, 57% of the chimeric mice receiving *Areg*^−/−^ BM died within several hours, and >80% died within 7 days. By contrast, no mice receiving WT-BMT died after PAB (Fig. 2d). AV block was recorded in the *Areg*^−/−^-BMT mice after PAB (Fig. 2e and Supplementary Table 3). In a publicly available single-cell RNA-seq dataset from heart stromal cells^13^, most *Areg*-expressing cells were *Cd68*^+^, indicating that macrophages are the major immune cell type expressing *Areg*, although some other cells might express AREG after PAB (Supplementary Fig. 6). Accordingly, the results of *Areg*^−/−^ BMT strongly suggest that AREG produced by macrophages is essential for protection against AV block and sudden death after PAB.

To further investigate whether AREG mediates the anti-arrhythmogenic action of macrophages, we next continuously administered AREG to clodronate-treated mice beginning the day prior of PAB. This AREG supplementation prevented the sudden death otherwise seen after PAB (Fig. 2f and Supplementary Table 3). Collectively, these results demonstrate that *Areg* deficiency confers abnormal cardiac electrical conduction and arrhythmogenicity, even in the steady-state, which leads to severe arrhythmias and sudden death after RV pressure overload.

It has been reported that sympathetic nerve activation triggers and facilitates arrhythmias and is an important mechanism underlying sudden cardiac death^14^, and that isoproterenol induces AV block and lethal arrhythmias in mice with conduction disturbances^15–17^. This prompted us to test the effects of β-adrenergic receptor stimulation. Isoproterenol administration (5 mg/kg) caused sudden death in ~57% of *Areg*^−/−^ mice within 2 days (Fig. 2g) and induced severe arrhythmias, including advanced AV block and ventricular fibrillation (VF) (Fig. 2h and Supplementary Fig. 7). All ECGs at the time of death showed VF in the isoproterenol-administered *Areg*^−/−^ mice. By contrast, WT mice had only infrequent isolated PVCs and did not die.

We further tested whether AREG deficiency promotes ventricular arrhythmogenic substrates by analyzing electrical activation in Langendorff-perfused whole heart preparations using optical mapping. VT/VF was induced through programmed stimulation or burst pacing. While VT was induced in only 1 of 6 WT hearts, all 6 *Areg*^−/−^ hearts developed VT when subjected to the VT/VF induction protocol (Fig. 2i).

### AREG regulates cardiac gap junction formation

**Fig. 3:**
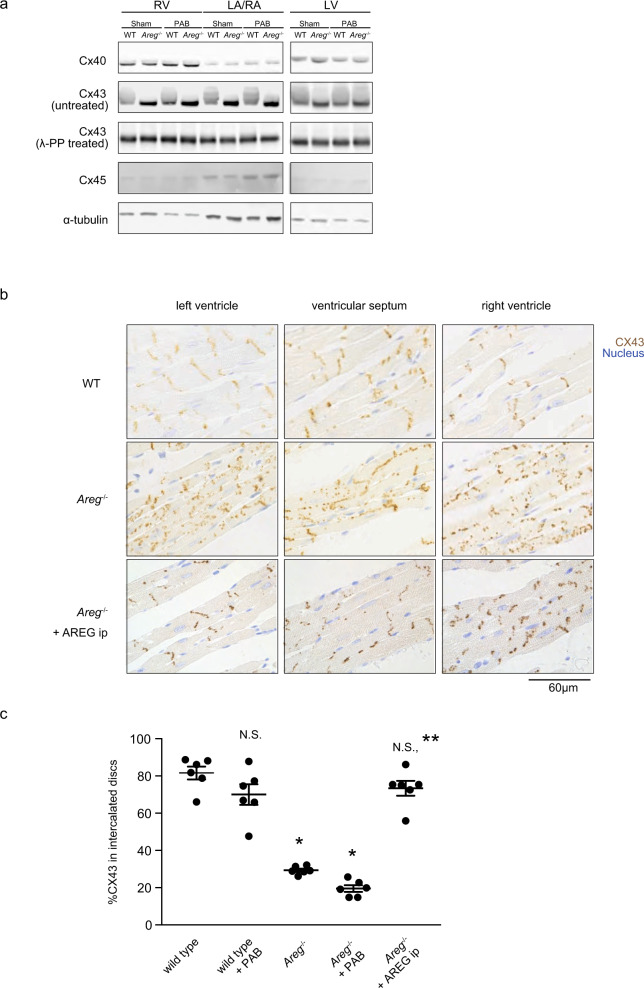
AREG promotes gap junctional connection between cardiomyocytes through Cx43. **a** Western blot analysis of Cx40, Cx43, and Cx45. Representative immunoblots are shown. α-Tubulin was used as a loading control. Note the mobility shift of Cx43 bands in WT mouse hearts. **b** Immunohistochemical staining of Cx43 (brown) in the myocardium. Nuclei (blue) were counterstained with methylene blue. WT and *Areg*^−/−^ mice were intraperitoneally administered either vehicle (PBS) or recombinant AREG (5 μg), and the hearts were harvested 30 min later. WT and *Areg*^−/−^ mice not receiving AREG were administrated vehicle (PBS) 30 min before sacrifice. The bar indicates 60 µm. The images show representative immunohistochemically stained hearts from WT or *Areg*^−/−^ mice at steady-state. **c** Fractions of Cx43 localized to the intercalated discs in cardiomyocytes in RVs. *n* = 6 mice in each group. One-way ANOVA followed by Tukey’s post-hoc test. *P < 0.0001 vs. WT. **P < 0.0001 vs. *Areg*^−/−^. Data are presented as mean values ± SEM.

Because *Areg*^*−/−*^ mice exhibited multiple types of arrhythmias, we speculated that they were subjected to global electrical conduction disturbances. Myocardial electrical conduction depends primarily on the intercellular transfer of current through gap junctions composed of connexin protein complexes^18,19^. We, therefore, performed western blotting to examine the cardiac expression of connexin (Cx) proteins in WT and *Areg*^−/−^ mice. Although there were no apparent differences in the levels of Cx40 and Cx45 or their band positions between WT and *Areg*^−/−^ mice, Cx43 bands from WT heart samples exhibited band shifts and smears, which were eliminated by treatment with lambda protein phosphatase before loading (Fig. 3a). This indicates that the band shifts and smearing reflect phosphorylation of Cx43. Levels of Cx43 phosphorylation appeared to be much reduced in *Areg*^−/−^ hearts, and the reduced phosphorylation persisted after PAB. Phosphorylation of Cx43 reportedly affects its localization and function^20^.

In mice, Cx43 is the primary component of gap junctions in both the atria and ventricles^21^. Phosphorylation of Cx43 is linked to its translocation and modulates gap junction properties, and dephosphorylation of Cx43 is associated with electrical uncoupling and susceptibility to arrhythmia^20,22^. We immunohistochemically stained Cx43 in mouse hearts (Fig. 3b). In WT mice, Cx43 was primarily localized to the intercalated discs between myocytes, as previously reported^23^. By contrast, the distribution of Cx43 was disorganized within cardiomyocytes in *Areg*^−/−^ mice, with Cx43 mislocated to the cells’ lateral surfaces (Fig. 3b, c and Supplementary Figs. 8 and 9). The disorganization was reversed by intraperitoneal administration of recombinant AREG (Fig. 3b). This mislocation of Cx43, called lateralization, is associated with arrhythmogenicity^24,25^. One consequence of Cx43 lateralization is the opening of Cx43 hemichannels that are mislocated away from intercalated discs and do not form gap junction channels with adjacent cells. This results in abnormal ion trafficking and electrical conduction^15,26^. To determine whether Cx43 lateralization is involved in the life-threatening arrhythmias seen in *Areg*^−/−^ mice, we utilized Gap19, which selectively inhibits Cx43 hemichannels without affecting normal gap junctional communication^17,27^. Intraperitoneal administration of Gap 19 to *Areg*^−/−^ mice clearly reduced the incidence of isoproterenol-induced death (Supplementary Fig. 10). These results suggest that AREG is critically involved in the regulation of Cx43 localization and cardiac gap junction formation, possibly through Cx43 phosphorylation, and that Cx43 lateralization is a cause of life-threatening arrhythmias triggered by cardiac stress in *Areg*^−/−^ mice.

### AREG promotes phosphorylation of Cx43

**Fig. 4:**
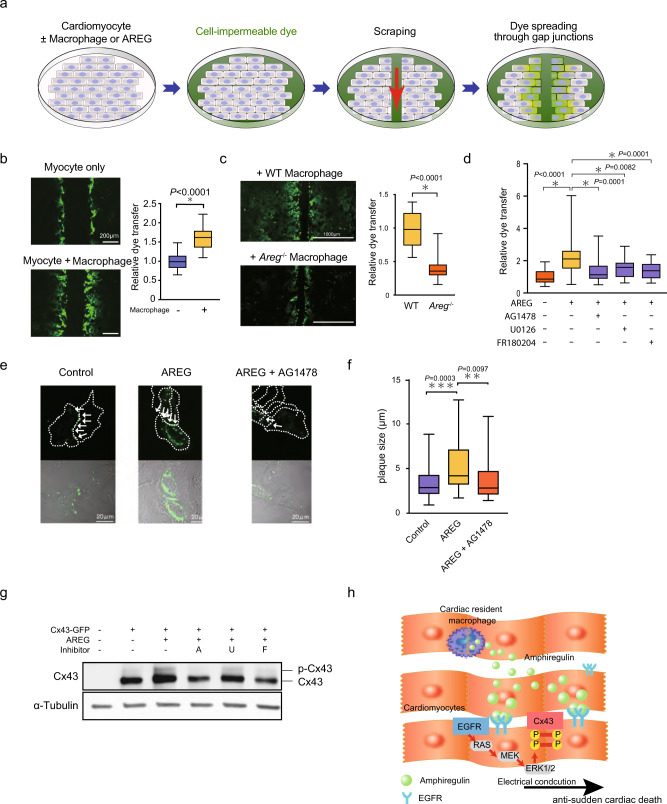
AREG facilitates gap junctional intercellular communication by stabilizing Cx43 gap junctions. **a** Schematic of the dye transfer assay. Mouse neonatal cardiomyocytes were cultured with or without cardiac resident macrophages. The cell-impermeable dye was loaded by scraping that caused tearing of the plasma membrane, and the level of dye transfer between cardiomyocytes was evaluated as the distance of dye-stained cells from the scraped edge after 15 min. **b**, and **c** Mouse neonatal cardiomyocytes were cultured with or without WT or *Areg*^*−/−*^ cardiac macrophages. Shown are representative images of dye-stained cells spreading from the scrape and the relative extent of dye transfer. Effects of macrophage coculture (**b**) and the lack of AREG in cocultured macrophages (**c**) on dye transfer were analyzed. *n* = 24 and 18, *n* = 22 and 23 (images) *P < 0.0001, two-tailed unpaired Student’s *t*-test. **d** Effects of AREG (100 ng/mL) and the indicated signal inhibitors (10 µmol/l, each) on dye transfer. *n* = 28, 28, 24, 24, and 30; *P < 0.05, post-hoc Dunnett’s test. **e**, **f** A *Cx43*-*GFP* plasmid was transfected into HeLa cells, and the cells were treated for 5 h with or without AREG (100 ng/mL) or AG1478 (10 µmol/l), as indicated. In **e**, formation of Cx43-GFP gap junction plaques (white arrows) at the cell-cell borders is shown. Cell borders are marked by dashed lines in upper panels. Scale bars, 20 µm. In **f**, plaque size is shown. *n* = 47, 39, and 36; **p < 0.01, ***p < 0.001, post-hoc Tukey’s test comparing cell adhesion between two-cardiomyocytes. **g** Western blotting of *Cx43-GFP* transfected HeLa cells treated for 6 h with AREG and 10 µmol/l AG1478 (A), U0126 (U), or FR180204 (F), as indicated. The positions of unphosphorylated and phosphorylated forms of Cx43 are shown. **h** Schematic model of how amphiregulin derived from cardiac macrophage suppresses arrhythmia. Throughout, box-and-whisker plots represent the median, the first and third quartiles, and the minimum and maximum values.

We next carried out dye transfer assays to further assess the effects of AREG on gap junctional intercellular communication (GJIC) between cultured mouse cardiomyocytes^28^. In this assay, a line is scraped into a confluent layer of cardiomyocytes, which enables a membrane-impermeant dye, 5(6)-carboxyfluorescein, to enter the affected cells through a transient tear in their plasma membrane. The dye can then migrate to adjacent cells through gap junctions. We quantified the level of dye transfer based on the distance the dye traveled from the scrape line (Fig. 4a). To assess the effects of cardiac macrophages on GJIC, we co-cultured cardiomyocytes with CD45^+^CD11b^+^Ly6G^-^F4/80^+^ Ly6C^lo^ cardiac macrophages. The level of dye transfer was significantly increased in the presence of macrophages (Fig. 4b).

We next tested the effects of AREG produced by macrophages on GJIC. As indicated by the dye transfer, GJIC between cardiomyocytes cocultured with *Areg*^*−/−*^ macrophages was significantly lower than between cardiomyocytes cocultured with WT macrophages (Fig. 4c). Moreover, recombinant AREG protein similarly increased the level of dye transfer (Fig. 4d). We then assessed GJIC function by using a cell motion imaging system to analyze the conduction velocity of contraction propagation in cultured cardiomyocytes. Conduction velocities in cardiomyocytes co-cultured with cardiac macrophages were higher than in cardiomyocytes cultured alone (Supplementary Fig. 11). AREG similarly increased conduction velocity in cultured cardiomyocytes. In mouse hearts in vivo, optical mapping showed that electrical propagation was impaired in *Areg*^−/−^ mice (Supplementary Fig. 12). These results indicate that AREG derived from cardiac macrophages facilitates functional coupling between cardiomyocytes and electrical propagation in the heart.

EGFR is reported to be a low-affinity receptor for AREG^29–31^. To test whether EGFR signaling mediates the effect of AREG on GJIC, we added an EGFR inhibitor (AG1478) or an inhibitor of MEK (U0126) or ERK (FR180204), two effectors downstream of EGFR. All three inhibitors significantly reduced the level of dye transfer mediated by AREG (Fig. 4c, d). This suggests that AREG secreted from cardiac macrophages induces GJIC between cardiomyocytes through EGFR and that AREG facilitates GJIC between cardiomyocytes at least in part via an EGFR/MEK/ERK pathway. To further elucidate how AREG-mediated phosphorylation of Cx43 enhances GJIC, we established HeLa cells expressing GFP-tagged Cx43 (Cx43-GFP) for live-cell imaging of Cx43. While HeLa cells do not endogenously express Cx43, exogenously expressed Cx43 does form functional gap junctions in these cells^32^. In the absence of AREG, Cx43-GFP was clustered in a few typical gap junction plaques between cells, which is consistent with the aforementioned report (Fig. 4e)^32^. After 5 h of AREG treatment, however, Cx43 formed much larger gap junctions at cell-cell contacts (Fig. 4e, f and Supplementary Fig. 13). The EGFR inhibitor AG1478 inhibited the formation of larger Cx43 plaques (Fig. 4e, f and Supplementary Fig. 13). Western blotting of the transfected cells showed that AREG induced phosphorylation of Cx43-GFP, which was blocked by inhibition of EGFR, MEK, or ERK. Thus, EGFR/MEK/ERK is a major pathway leading to Cx43 phosphorylation (Fig. 4g). However, Cx43 is known to be phosphorylated at multiple sites by multiple signals^33,34^. To detect additional AREG-mediated pathways leading to Cx43 phosphorylation, we mutated the target phosphorylation sites on Cx43-GFP that correspond to the Akt, PKC, PKA, CK1, and MAPK signaling pathways. The results showed that the sites corresponding to PKA, CK1, and MAPK are required for the full phosphorylation of Cx43 induced by AREG (Supplementary Fig. 14). Additional mutant analysis of phosphorylation sites targeted by MAPKs revealed the MEK/ERK pathway may be essential for Cx43 phosphorylation (Supplementary Fig. 14).

Among EGFR ligand genes, including *Areg*, *Hbegf*, *Tgfa*, *Egf*, *Ereg*, *Btc*, and *Epg*, cardiac macrophages expressed only *Areg* and *Hbegf* (Supplementary Fig. 15). While AREG promoted phosphorylation of Cx43, HBEGF did not (Supplementary Fig. 14). Similarly, while AREG increased the conduction velocity of cardiomyocyte contraction, HBEGF did not (Supplementary Fig. 11). These results show that the two EGFR ligands produced by cardiac macrophages exhibit differential actions in cardiomyocytes. Taken together, these findings indicate AREG induces Cx43 phosphorylation via an EGFR/MEK/ERK pathway, which facilitates formation of stable gap junctions, thereby promoting intercellular communication (Fig. 4h).

## Discussion

Cardiac arrhythmias are a primary contributor to sudden cardiac death in patients with heart disease^35^ and preventing sudden cardiac death remains a major unmet medical need. It has been suggested that altered expression of various genes, including genes related to ion channels and gap junctions^36,37^, contributes to the arrhythmogenicity in heart failure patients. The present study demonstrates that AREG derived from cardiac macrophages maintains electrical impulse conduction by controlling the alignment of Cx43 at gap junctions between cardiomyocytes. Phosphorylation of Cx43 by AREG facilitates intramyocardial conduction by promoting formation of large gap junctions. Previous studies showed that in a normal heart, Cx43 proteins primarily exist in a phosphorylated state and that ischemia induces dephosphorylation^20^. Our finding that gap junctions are disorganized in *Areg*^*−/−*^ mice suggests that dysregulation of Cx43 phosphorylation is an important mechanism underlying severe arrhythmia and sudden death induced by RV stress. That said, functions other than Cx43 regulation, such as regulatory roles in other processes in cardiac physiology and stress response^5^, may also be involved in AREG’s antiarrhythmic action.

Hulsmans et al. recently reported that macrophages contribute to cardiac conduction in the AV node by directly connecting to cardiomyocytes through Cx43-containing gap junctions^38^. While *Cx43* deletion from macrophages prolonged AV conduction, it did not cause advanced AV block, suggesting that additional mechanisms, such as AREG-mediated facilitation of intramyocardial electrical coupling, are critically involved in AV nodal conduction^14^. More importantly, cardiac macrophages exist throughout the myocardium, and our finding that *Areg* deletion generates substrates for ventricular arrhythmia suggests that macrophages and AREG are required for maintenance of myocardial electrical conduction widely throughout the heart.

In addition to systemic and hematopoietic loss of *Areg*, acute induction of macrophage damage using clodronate liposomes made mice highly susceptible to severe arrhythmia and sudden death. This indicates that macrophages are required not only to establish gap junctional communication, but also to continuously and dynamically regulate gap junctions, particularly during cardiac stresses, such as acute RV pressure overload. AREG supplementation prevents sudden death, which also highlights the dynamic nature of gap junctional regulation and suggests that cardiac macrophages and AREG are potential therapeutic targets for both long-term and acute treatment of life-threatening arrhythmias. Notably, Son et al. reported that *Areg* expression is reduced in LV myocardial tissues of patients who suffered sudden cardiac death^39^, suggesting that AREG is also important for preventing severe arrhythmias in humans.

## Methods

### Animal studies

Male C57BL6/J mice were purchased from CLEA Japan and Jackson Laboratory and maintained on a standard mouse chow diet under sterile barrier conditions, on a 12-h light-dark cycle with 18–23 °C and 40–60% humidity. Male *Areg* homozygous null (*Areg*^−/−^) mice were backcrossed to C57BL6/J mice over ten generations^5^. *Rag2*^−/−^, *Cd4*^−/−^, and *Cd8a*^−/−^ mice were purchased from Taconic (Germantown, NY). The genotypes of all mice were determined using genomic PCR. The isoproterenol challenge entailed bolus administration of isoproterenol (Sigma) 5 mg/kg intraperitoneally under constant ECG recording^17^. Clodronate liposomes and control liposomes were purchased from LIPOSOMA B.V. A total of 10 μl of clodronate or control liposome solution per gram of mouse were intravenously administrated 24 h before PAB. After the first administration of clodronate or control liposomes, the same volume of liposomes was also administrated every 7 days for long term depletion, per the manufacturer’s instructions. All protocols for animal experiments were approved by the Animal Care and Use Committee of the University of Tokyo.

### Cell culture

Mouse cardiomyocyte cultures were prepared from one- to three-day-old male and female C57BL6/J mouse pups using a neonatal heart dissociation kit (Miltenyi Biotec GmbH, Bergisch Gladbach, Germany). Cardiac macrophages were depleted from primary cultures of cardiomyocytes using anti-CD11b microbeads (Miltenyi Biotec GmbH, Bergisch Gladbach, Germany) with an autoMACS® Pro Separator according to the manufacturer’s instructions.

The separated cells were maintained in a 5% CO_2_ incubator at 37 °C on collagen-coated 35-mm culture dishes in high glucose Dulbecco’s modified Eagle’s medium (DMEM; Gibco, 11995-065, USA) supplemented with 10% fetal bovine serum (FBS; HyClone, SH30396.03, USA), 100 IU/ml penicillin and 100 μg/ml streptomycin. The cell cultures were used for scrape loading/dye transfer assays when they reached confluence.

Human epithelioid cervix carcinoma cells (HeLa cells, ATCC, CCL-2) were cultured in DMEM (Gibco, 11995-065, USA) supplemented with 10% FBS, 100 U/ml penicillin, 100 μg/ml streptomycin, and 2 mmol/l glutamine. The cells were split the day before transfection at 70–90% confluency. The cells were transfected using LipofectAMINE 2000 (Life Technologies, 11668-019, USA) according to the manufacturer’s instructions. A total of 10 µmol/l AG1478 (Wako, 017-20151, Japan), UO126 (Abcam, ab120241, USA) or FR180204 (Sigma, SML0320, USA) was added 1 h before supplementation with the indicated concentrations of recombinant mouse AREG protein (989-AR/CF, R&D systems, Minneapolis, MN, USA). The transfected cells were visualized using confocal microscopy (LSM 5100 Meta, Carl Zeiss, Germany). For analysis of the effects of EGFR ligands on Cx43 phosphorylation (Supplementary Fig. 3), AREG (100 ng/mL), HBEGF (10 ng/mL, SRP6050, Sigma, USA), or TGF-α (100 ng/mL, 239-A-100, R&D systems, Minneapolis, MN, USA) were added to the cells for 30 min.

### Scrape loading/dye transfer assay

GJIC levels were determined using the scrape loading/dye transfer technique as previously reported, with minor modifications^28^. Briefly, cardiomyocytes were grown to confluence in 35-mm culture dishes. For coculture with cardiac-resident macrophages, sorted cardiac CD11b^+^F4/80^+^Ly6C^lo^Ly6G^-^ macrophages (15,000 cells/per well) were added to cardiomyocytes and cultured for 24 h. The cells were scraped in phosphate buffered saline (PBS) containing 1 mmol/l 5(6)-carboxyfluorescein, which can pass through gap junctions. Three min after the scraping, the cells were washed with PBS, and fluorescence images were captured using a EVOS FL auto cell imaging system (ThermoFisher Scientific, Waltham MA, USA). The distance the dye spread from the border of the scratch was measured. At least five images per well were used to assess the extent of the dye transfer. For tests of AREG and signal inhibitors, AREG (100 ng/mL) and AG1478, U0126, or FR180204 (10 µmol/l) were added to the cells and incubated for 30 min before scraping.

### Plasmid construction

To construct a green fluorescent protein (GFP)-tagged Cx43 expression plasmid, P132 and Cx43 delta dt GFP were purchased from Cecilia Lo (Addgene Plasmid, USA, #17668 and #17671). P132 was digested with KpnI and BpuI, and Cx43 delta dt GFP was digested with KpnI and XbaI. Oligonucleotides were used to link the full-length *Cx43* and the *Gfp*-containing backbone. After ligation using T4 DNA ligase (TKR, 6023, Japan), competent DH5α *Escherichia coli* (TKR, 9057, Japan) was transformed with the plasmid, positive colonies were identified and selected, and the insert size was confirmed by gel electrophoresis. Thereafter, the sequence of the cDNA encoding the chimeric protein was verified. The sequence of the oligonucleotides used in these experiments are shown in Supplemental Table 4.

To investigate which amino acids in Cx43 are phosphorylated by AREG, we used a QuikChange Lightning Multi Site-Directed Mutagenesis Kit (Agilent Technologies, California, USA) according to the manufacturer’s instructions to introduce mutations at the consensus phosphorylation sites for the Akt (S373), PKC (S368), cAMP (S365), CK1 (S325/328/330) and MAPK (S255/262/279/282) pathways by substituting the serine residues of Cx43-GFP with alanine. The sequences of the primers used were shown in Supplemental Table 4.

### Mouse model preparation

For the BMT experiments, 8-week-old mice were irradiated with a lethal dose (9 Gy). After 24 h, BM cells (1 × 10^6^) isolated from donor mice were injected into the recipient mice through the tail vein. The donor BM was harvested in DMEM, filtered through a 100 μm cell strainer (Greiner Bio-One, 542000, Austria), washed in fresh DMEM with 10% FBS, and resuspended in PBS.

For the PAB experiments, the surgery was performed largely as previously described^40^. We optimized procedures to minimize perioperative death and gain consistent RV pressure overload. Briefly, 8- to 12-week-old mice were anesthetized with intraperitoneal urethane, after which anesthesia was maintained with 2% isoflurane. After orotracheal intubation, the mice were mechanically ventilated. An incision was made in the second left intercostal space using sterile techniques. The pulmonary artery was isolated, and a suture ligature was placed around the vessel. The suture was tied against a 23-gauge needle that was then rapidly removed. After the chest was closed, the mouse was extubated and allowed to recover from the anesthesia. Control mice underwent sham thoracotomies. The RV pressure was around 30 mmHg after PAB when measured in several mice using a pressure catheter (FTH-1211B-0018, Primetech Corporation, Japan).

For repeated AREG administrations to mice, 5 μg recombinant mouse AREG protein (989-AR/CF, R&D systems, Minneapolis, MN, USA) dissolved in 50 μl of PBS was intraperitoneally administrated to *Areg*^−/−^ mice 3 times a week, as previously described^5^. For continuous administration of AREG to mice, 5 μg/day of AREG was administrated using osmotic minipumps (Alzet, Cupertino, CA, USA).

### Immunohistochemistry

Cx43 staining was accomplished using anti-Cx43 antibodies (C6219, Sigma, 1:2000) and detected using a DAB system (R&D Systems, Minneapolis MN, USA). The images were acquired using a FSX1000 (Olympus, Tokyo, Japan). Quantification of Cx43 present at gap junctions was evaluated by measuring the area of Cx43 at gap junctions and total Cx43 using Image J 1.53a software (NIH, USA).

Mice were perfused with PBS and then fixed overnight in a formalin- and methanol-based fixative solution (Ufix, Sakura Fine Tech, Japan). The following day, the fixed tissues were dehydrated and embedded in paraffin. To quantify cardiac fibrosis, we stained heart sections with Picrosirius red.

Paraffin-embedded fixed heart tissues were cut into 5-µm-thick sections, after which the sections were dewaxed, the antigens were retrieved using microwave antigen retrieval (pH 8.0), and the sections were incubated first with mouse anti-N-cadherin antibody (33-3900, ThermoFisher, USA, 1:500) and rabbit anti-CX43 antibody (C6219, Sigma, USA, 1:2000), and then with Alexa-Fluor-488-conjugated anti-mouse-IgG (A-11001, ThermoFisher, USA, 1:2000) and Alexa-Fluor-635-conjugated anti-rabbit-IgG (A-31576, ThermoFisher, USA, 1:2000). The heart sections were visualized using confocal microscopy (LSM 5100 Meta, Carl Zeiss, Germany).

### Western blotting

Hearts were removed from the experimental mice and flash-frozen in liquid nitrogen. The tissues were minced using a BioMasher® II (Nippi, 300-95441, Tokyo, Japan), after which the cell pellets were lysed with RIPA buffer containing a protease inhibitor cocktail (COMPLETE, EDTA-free, Roche, USA). After protein concentrations were determined using a BCA protein assay kit (Pierce, 23227, Rockford IL, USA), protein samples were separated using SDS-PAGE and transferred to nitrocellulose membranes (BioRad, 1620112, Hercules CA, USA). In the dephosphorylation experiments, the samples were treated for 1 h with lambda protein phosphatase (1U/μl, sc-200312A, Santa Cruz Biotechnology, USA) before being loaded onto the gel. The following primary antibodies were used for western blotting: rabbit anti-Cx40 polyclonal antibodies (AB1726, Merck Millipore, USA, 1:500), rabbit anti-Cx43 polyclonal antibodies (C6219, Sigma, USA, 1:2000) and rabbit anti-Cx45 polyclonal antibodies (AB1745, Merck Millipore, USA, 1:2000). Mouse anti-α-tubulin monoclonal antibodies (T6199, Sigma, USA, 1:1000) were used to normalize expression. Secondary goat anti-rabbit IgG-HRP antibodies (Cell Signaling Technology, 7074, USA, 1:5000) or goat anti-mouse IgG-HRP antibodies (Cell Signaling Technology, 7076, USA, 1:5000) were used as probes, and peroxidase activity was detected using Clarity™ Western ECL blotting substrate (Bio-Rad, 170-5061, USA) and an ImageQuant™ LAS 4000mini (GE Healthcare, Piscataway NJ, USA).

### Long-term ECG monitoring

Long-term ECGs were recorded from experimental mice using implanted telemetry devices (Softron, Tokyo, Japan). Under intraperitoneal anesthesia, a radiofrequency transmitter was inserted into a subcutaneous tissue pocket. The leads were tunneled subcutaneously and fixed to the pectoral muscle in the lead II configuration. The experiments were performed 1 week after the surgical instrumentation. The telemetry data were recorded continuously through a receiver placed under the mouse cage. LabChart pro version 8 (AD Instruments, Australia) was used for beat-to-beat analysis to detect arrhythmias manually. VT/VF were diagnosed based on Lambeth Convention guidelines^41^.

### Optical action potential mapping

We used a high-resolution optical mapping system to examine the electrophysiological properties of the heart. Details of the system and the mapping procedure were described previously^42^. In brief, isolated mouse hearts were continuously perfused on a Langendorff apparatus with modified Krebs-Ringer solution equilibrated with 95% O_2_-5% CO_2_ (37 °C, pH 7.4). The hearts were then stained with the voltage-sensitive dye 4-{β-[2-(di-*n*-butylamino)-6-naphthyl]vinyl}pyridinium (di-4-ANEPPS). We used premature stimulation or burst pacing to induce VT/VF. After rapid stimulation at an S1-S1 interval of 100 ms, a single premature stimulation (S2) was applied, and the sequence was repeated with progressive shortening of the S1–S2 interval. When VT/VF was not induced, burst pacing was applied. In addition, VT/VF induction was also tested in the same protocol under 3 µM isoproterenol.

Conduction velocity (CV) was measured as previously described^43^. Briefly, CV was measured during constant stimulation of the center of the LV free wall using a basic cycle length of 150 ms. The longitudinal (L) direction of propagation was determined from the activation map so that it crossed the most widely spaced isochrones. A second line for the transverse (T) direction of propagation was drawn perpendicular to the first line through the most densely spaced isochrone.

### Echocardiography

Mice were anesthetized with isoflurane (2–3% for induction; 0.25–2% for maintenance) and set in a supine position. Transthoracic echocardiography was recorded using a Vevo 2000 imaging system (VisualSonics). Pulmonary artery peak velocity, right ventricular fractional shortening, and right ventricular wall-thickness were measured as previously reported^44^.

### Flow cytometric analysis and cell sorting

Samples for flow cytometry were prepared as previously described^5^. Briefly, mice were anesthetized, and the heart was exposed and perfused with 10 ml PBS from the left ventricle. Thereafter, the heart tissue was mechanically minced using a scalpel, and the tissue was incubated in Dulbecco’s modified Eagle’s medium (DMEM) containing 1% elastase (Worthington Biochemical) for 120 min at 37°C. During the incubation, the cells in the suspension were dissociated by sequentially passing the extract through 20-, 21- and 23-gauge needles at 30-min intervals. To further dissociate the cells, they were passed through a 23-gauge needle three times and then filtered through a 40-µm cell strainer (BD). The cells were then centrifuged at 300 × *g* for 5 min, washed with PBS, and resuspended in FACS buffer (PBS supplemented with 1% FBS). After removing erythrocytes in BD PharmLyse solution (BD), the isolated cells were stained with fluorochrome-conjugated antibody and subjected to flow cytometric analysis and cell sorting using a FACS Aria III instrument (BD). Data were acquired on FACSDIVA v8.0.2 (BD) and analyzed using FlowJo (Tree Star). Cells isolated for RNA purification were fixed before staining with antibodies for flow cytometric analysis, as previously reported^45^. FACS-sequential gating strategies were shown in Supplementary Figure 16.

### Antibodies

For flow cytometric analysis, anti-CD45.2-V500(562129, BD bioscience, 1:100), anti-CD45.2-APC(109814, BioLegend, 1:100), anti-CD4-PerCP/Cy5.5(100433, BioLegend, 1:100), anti-CD8a-PerCP/Cy5.5(100733, BioLegend, 1:100), anti-CD11b-PerCP/Cy5.5(45-0112-80, eBioscience, 1:100), anti-Ly6g-PerCP/Cy5.5(127615, BioLegend, 1:100), anti-B220-PerCP/Cy5.5(103235, BioLegend, 1:100), anti-CD11b-BV421(101251, BioLegend, 1:100), anti-CD64-APC(139306, BioLegend, 1:100), anti-F4/80-PE(123110, BioLegend, 1:100), anti-Ly6c-PE-Cy7(128018, BioLegend, 1:100) were used. For western blotting, anti-Cx40(AB1726, Merck Millipore, 1:500), anti-Cx43(C6219, Sigma, 1:2000), anti-Cx45(AB1745, Merck Millipore, 1:2000), anti- α -tubulin(T6199, Sigma, 1:1000), anti-rabbit IgG-HRP(7074, Cell Signaling Technology, 1:5000), anti-mouse IgG-HRP(7076, Cell Signaling Technology, 1:5000) were used. For immunohistochemistry, anti-F4/80(MCA497G, Serotec, 1:500), anti-Ly6G(127601, BioLegend, 1:500), anti-B220 (103201, BioLegend 1:500), anti-CD3(GTX42110, GeneTex, 1:500), anti-Cx43(C6219, Sigma, 1:2000), anti-N-cadherin(33-3900, ThermoFisher, 1:500) were used. The second antibodies used were Alexa-Fluor-488-conjugated anti-mouse-IgG(A-11001, ThermoFisher, 1:2000) and Alexa-Fluor-635-conjugated anti-rabbit-IgG(A-31576, ThermoFisher, 1:2000).

### Video microscopy and propagation analysis of cultured cardiomyocytes

Video images of cultured cardiomyocytes were recorded as sequential phase-contrast images with a 4x objective at a frame rate of 150 frames/s and a resolution of 2048 × 2048 pixels using a SI8000 cell motion imaging system (Sony Corporation, Tokyo, Japan). The conduction velocity of the excitation wave was calculated by the SI8000 system. Cardiomyocytes were harvested from neonatal mouse hearts using Neonatal Cardiomyocyte Isolation Kit, mouse (Miltenyi Biotec). All isolated cardiomyocytes from 14 murine hearts of neonates aged from postnatal day 0 were equally divided into all wells of one 48 wells-dish. After the three days-culture with Dulbecco’s Modified Eagle Medium (Gibco, Thermo Fisher SCIENTIFIC) containing 10% fetal bovine serum (GE HealthcareHyClone), L-glutamine, Penicillin and Streptomycin, cardiomyocytes reached confluent. AREG was supplemented after 10 h. serum starvation.

### Real-time PCR analysis

Total RNA was purified from cells using a RNeasy plus micro kit (Qiagen) according to the manufacturer’s instructions. Quantitative real-time PCR analyses were carried out using a Lightcycler 480 system (Roche), with 18 S rRNA serving as an internal control. The sequences of the primers used were shown in Supplemental Table 4.

### Single-cell transcriptome analysis

FASTQ data (E-MTAB-7376)^13^ were downloaded from Array Express and processed using 10xGenomics Cell Ranger count software and Seurat version 3^46^. We filtered out cells that expressed fewer than 200 unique molecular identifiers (UMI), and the percentage of counts mapped to the mitochondrial genome was ≥5%. To exclude possible doublets, cells with high UMI counts were also excluded. Datasets (sham of total interstitial cells and *Pdgfra*^+^ cells) were aggregated using Seurat’s standard integration procedure with 2000 highly variable genes. The expression matrix was dimensionally reduced using principal component analysis of the corrected integrated gene matrix. Clusters were identified using a graph-based approach with the Louvain modularity optimization algorithm. We employed i-SNE for dimensionality reduction and visualization of our datasets. To view gene expression, the MAGIC algorism was used to impute drop-out values^46^.

### Statistics and reproducibility

Data are presented as box-and-whisker plots showing the median, the first and third quartiles, and the minimum and maximum values. The data were analyzed using either an unpaired Student’s *t*-test or ANOVA followed by Dunnett’s, Tukey’s, or Holm–Sidak multiple comparison test, as indicated. Kaplan–Meier analysis followed by a log-rank test with or without post hoc Bonferroni’s correction was used to compare survival between mouse groups. The frequencies of VT/VF induction were compared using the χ^2^ test. All the statistical analyses were performed using Microsoft Excel 2019 and GraphPad Prism 8 software (GraphPad Software, Inc., San Diego, CA, USA). Each experiment was repeated independently at least 2–3 times with similar results.

### Reporting summary

Further information on research design is available in the Nature Research Reporting Summary linked to this article.

## Supplementary information

Supplementary Information

Reporting Summary

## Data Availability

All data supporting the findings of this study are available from the corresponding author upon reasonable request. All source data are provided. Source data are provided with this paper.

## Source data

Source Data
