# Supplementary material for: Cardiac macrophages prevent sudden death during heart stress
Source: Nat Commun. 2021 Mar 26;12:1910. doi: 10.1038/s41467-021-22178-0 (PMC7997915; doi:10.1038/s41467-021-22178-0)

Supplementary Information

Cardiac Macrophages Prevent Sudden Death During Heart Stress

Junichi Sugita[1], Katsuhito Fujiu[1,2,3*], Yukiteru Nakayama[1], Takumi Matsubara[1], Jun Matsuda[1], Tsukasa Oshima[1], Yuxiang Liu[1], Yujin Maru[1], Eriko Hasumi[1], Toshiya Kojima[1], Hiroshi Seno[4], Keisuke Asano[4], Ayumu Ishijima[4], Naoki Tomii[4], Masatoshi Yamazaki[4], Fujimi Kudo[5], Ichiro Sakuma[4], Ryozo Nagai[6], Ichiro Manabe[5*] and Issei Komuro[1]

[1]Department of Cardiovascular Medicine and [2]Department of Advanced Cardiology, the University of Tokyo, [3]PRESTO, Japan Science and Technology Agency, [4]Medical Device Development and Regulation Research Center, Department of Bioengineering/Department of Precision Engineering, School of Engineering, The University of Tokyo Department of Disease Biology and Molecular Medicine, [5]Department of Disease Biology and Molecular Medicine, Graduate School of Medicine, Chiba University, [6]Jichi Medical University

*Address for correspondence

Katsuhito Fujiu or Ichiro Manabe

e-mail: fujiu-tky@umin.ac.jp or manabe-tky@umin.ac.jp

7-3-1 Hongo, Bunkyo, Tokyo, Japan

Supplementary Table 1 AV block occurrence after the PAB experiments corresponding to Figure 1c

Mice	AVB undetected	AVB detected	AVB frequency (%)
Control mice for Clodronate	12	0	0
Clodronate	3	4	57.1
Control WT for Ly6G Ab	10	0	0
Ly6G Ab	10	0	0
Control WT for CD4KO	10	0	0
CD4KO	10	0	0
Control WT for CD8KO	10	0	0
CD8KO	10	0	0
Control WT for *Rag2* KO	10	0	0

Numbers of mice and frequencies of AV block (AVB) are shown. ECGs were recorded before and after PAB every 1 h up to 8 h after PAB in all mice. After that, ECGs were checked once a day. If mice showed any abnormal symptoms, ECGs were taken to detect arrhythmias. AVB was defined as progressive AVB that resulted in sudden death with no ventricular escape.

Supplementary Table 2 Arrhythmia occurrence in mice shown in Figure 2a

Mice	PAC	PVC	AVB	Sinus arrest/ SA block
WT	0.00 ± 0.00	0.00 ± 0.00	0.00 ± 0.00	0.00 ± 0.00
Areg$^{-/-}$	0.33 ± 0.52	6.50 ± 2.51**	28.33 ± 5.05**	1.83 ± 0.75*

Mean numbers ± S.D of each arrhythmia observed in each mouse during 24 h. ECGs were continuously recorded by telemetry for 24 h under free moving. AVB includes second and third-degree AVB. No life-threatening AVB was documented in these recordings. n=6 in each group. *P<0.001, ***P<0.0001, two-tailed unpaired Student's t-test. PAC: premature atrial contraction, PVC: premature ventricular contraction.

Supplementary Table 3 AV block occurrence after the PAB experiments corresponding to Figure 2b-f

Mice	AVB undetected	AVB detected	AVB frequency (%)
WT	10	0	0
Areg$^{-/-}$	1	6	85.7
WT-BMT	9	0	0
Areg$^{-/-}$-BMT	1	6	85.7
Clodronate+PBS	2	8	80
Clodronate+AREG	9	1	10

ECGs were recorded and evaluated as described in Supplementary Table 1.

Supplementary Table 4 primer sequences

primer	Sequence
sense oligonucleotide for *Cx43*	5’-TTAGCTATCGTGGATCAGCGACCTTCCAGCAGAGCCAGCAGCCGCGCCAGCAGCAGACCTCGGCCTGATGACCTGGAGATTT-3’
anti-sense oligonucleotide for *Cx43*	5’-CTAGAAATCTCCAGGTCATCAGGCCGAGGTCTGCTGCTGGCGCGGCTGCTGGCTCTGCTGGAAGGTCGCTGATCCACGATAGC-3’
Cx43 S373A	5’-GGCCGAGGTCTGGCGCTGGCGCGGCT-3’
Cx43 S368A	5'-GCTGGCGCGGCTGGCGGCTCTGCTGGAA-3'
Cx43 S365A	5'-GGCTGCTGGCTCTGGCGGAAGGTCGCTGAT-3'
Cx43 S325/328/330A	5’-GGGCGTGGGCGTTGGCGATGGTGGCTCCGGCCTGCCC-3'
Cx43 S255/262A	5'-CGGCCCACTGGCCCCATCCAAAGACTGCGGAGCTCCAAAATATGC-3'
Cx43 S279/282A	5’-CGGCCCCACTCGCACCTATGGCTCCTCCTGGG-3'
Areg up	5’-AAGAAAACGGGACTGTGCAT-3’
Areg down	5’-GGCTTGGCAATGATTCAACT-3’
Hbegf up	5’-TCTTCTTGTCATCGTGGGACT-3’
Hbegf down	5’-CACGCCCAACTTCACTTTCT-3’

Supplementary Figure 1. RV Fibrosis and immune cell infiltration 3 days after PAB

a, Representative picrosirius red stained section of a WT mouse heart 3 days after PAB. Bar, 1 mm. Note that fibrosis is apparent only in the RV wall. Arrowheads indicate the fibrotic area. b-e, Representative immunohistochemical staining for the indicated markers in the RV of WT hearts 3 days after PAB. Immune cells, including F4/80$^+$ macrophages (b), Ly6G$^+$ neutrophils (c), B220$^+$ B cells (d), and CD3$^+$ T cells (e), accumulated mainly in the myocardium and epicardium of the RV. Bars, 250 µm.

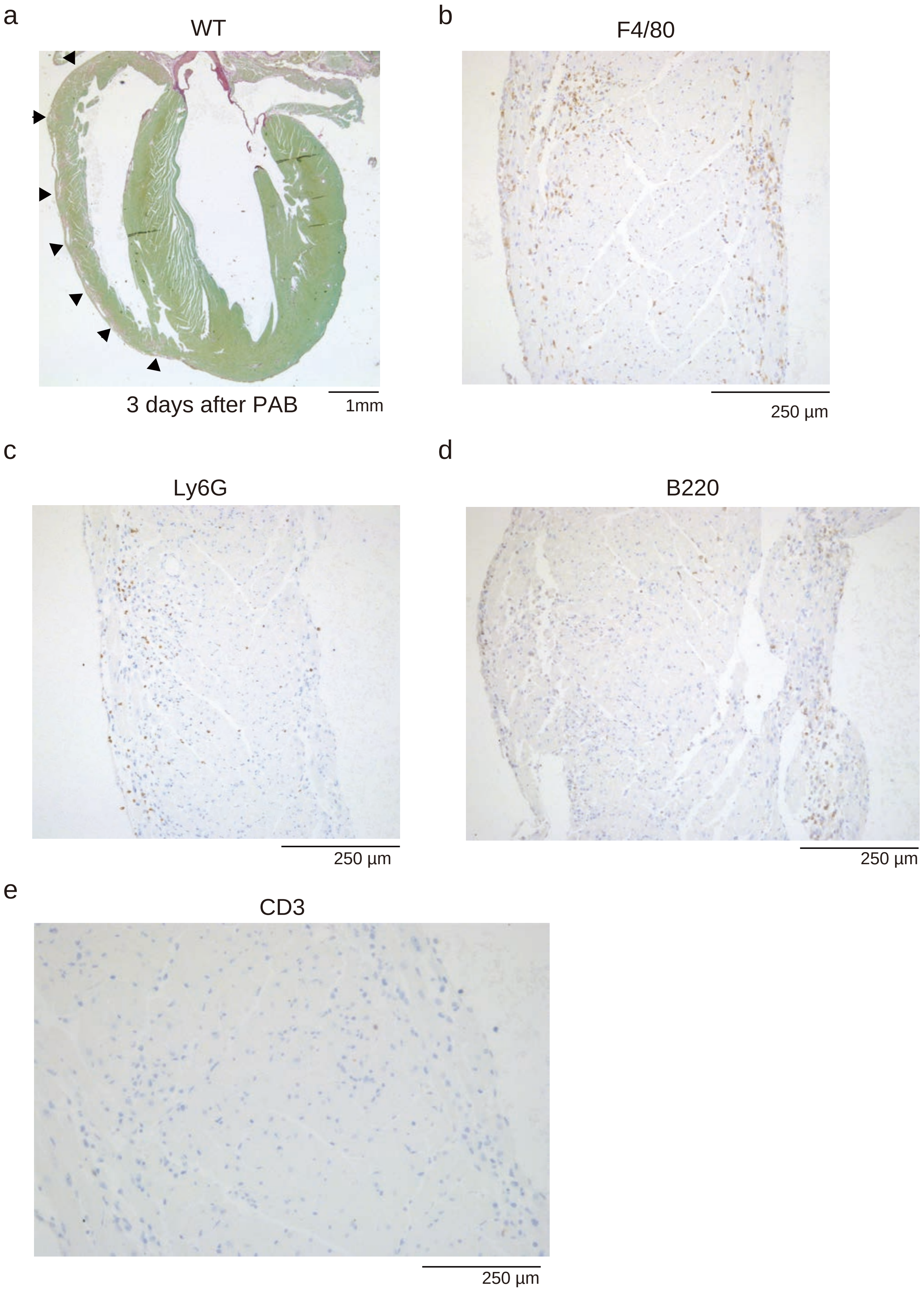
a
WT
3 days after PAB
1mm
b
F4/80
250 µm
c
Ly6G
250 µm
d
B220
250 µm
e
CD3
250 µm

Supplementary Figure 2. Impact of PAB on RV pressure overload

Cx3cr1$^{CreER/+}$;*R26*$^{iDTR/+}$ mice were treated with tamoxifen, which induced CX_3CR1^+ cell-specific DTR expression[1], after which they were treated with diphtheria toxin to deplete CX_3CR1^+ cells. *Cx3cr1*$^{CreER/+}$;*R26*$^{+/+}$ mice were used as control macrophage-intact mice. On the day after the second administration of diphtheria toxin, mice were subjected to PAB. The experimental schedule is shown schematically. The PA peak velocity was recorded from echocardiography with a parasternal short-axis view at the level of the aortic valve 15 minutes after PAB. *$P<0.05$ vs. non-PAB mice receiving the same macrophage treatment. One-way ANOVA followed by Tukey's post-hoc test. n=6 in each group, except macrophages in the intact-PAB group (n=18). Data are presented as mean values ± SEM.

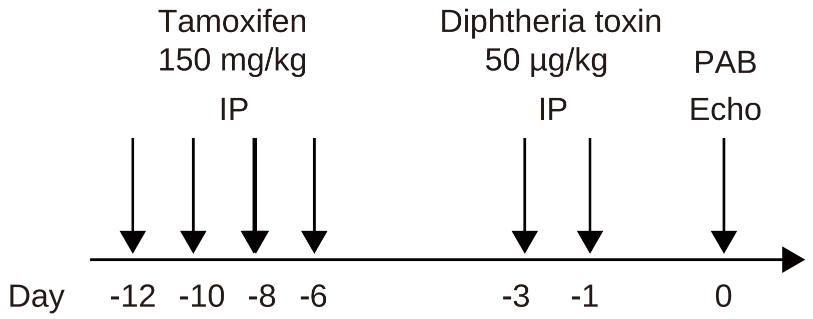
Tamoxifen
150 mg/kg
IP
Diphtheria toxin
50 µg/kg
IP
PAB
Echo
Day
-12
-10
-8
-6
-3
-1
0

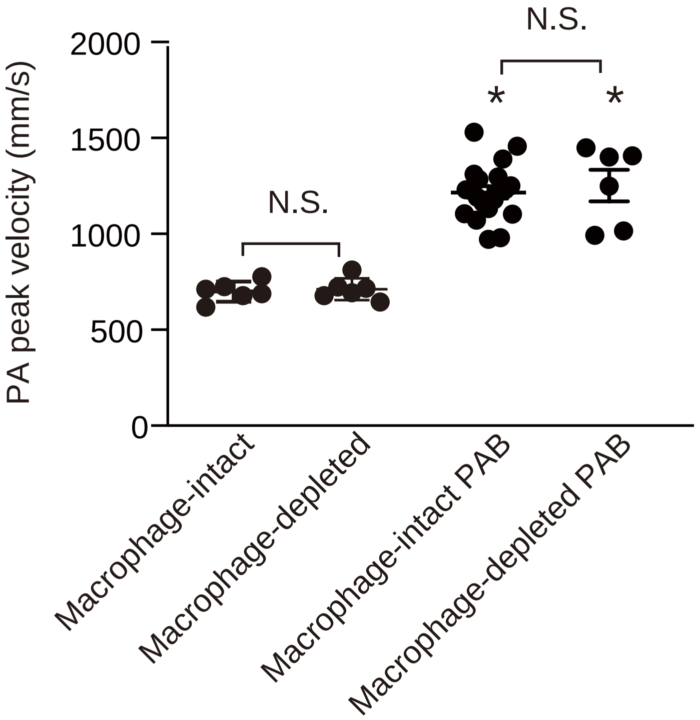
PA peak velocity (mm/s)
2000
1500
1000
500
0
N.S.
N.S.
*
*
Macrophage-intact
Macrophage-depleted
Macrophage-intact PAB
Macrophage-depleted PAB

Supplementary Figure 3. Effects of PAB in macrophage-depleted *Cx3cr1*$^{CreER/+}$;*R26*$^{iDTR/+}$ mice

a, Flow cytometric count of cardiac-resident macrophages (CD45^{+}F4/80^{+}CD11b^{+}Ly6G^{-}Ly6C^{-} cells) in *Cx3cr1*$^{CreER/+}$;*R26*$^{iDTR/+}$ mice 3 days after administration of diphtheria toxin. ***P<0.01, n=6 in each group, two-tailed unpaired Student *t*-test. *Cx3cr1*$^{CreER/+}$;*R26*$^{+/+}$ mice treated with tamoxifen and diphtheria toxin were used as a control. Data are presented as mean values ± SEM. b, Survival rates after PAB. *P<0.05, Log-rank test. c, Representative ECG recorded just before the death of a *Cx3cr1*$^{CreER/+}$;*R26*$^{iDTR/+}$ mouse that had undergone PAB. The arrow heads indicate P waves.

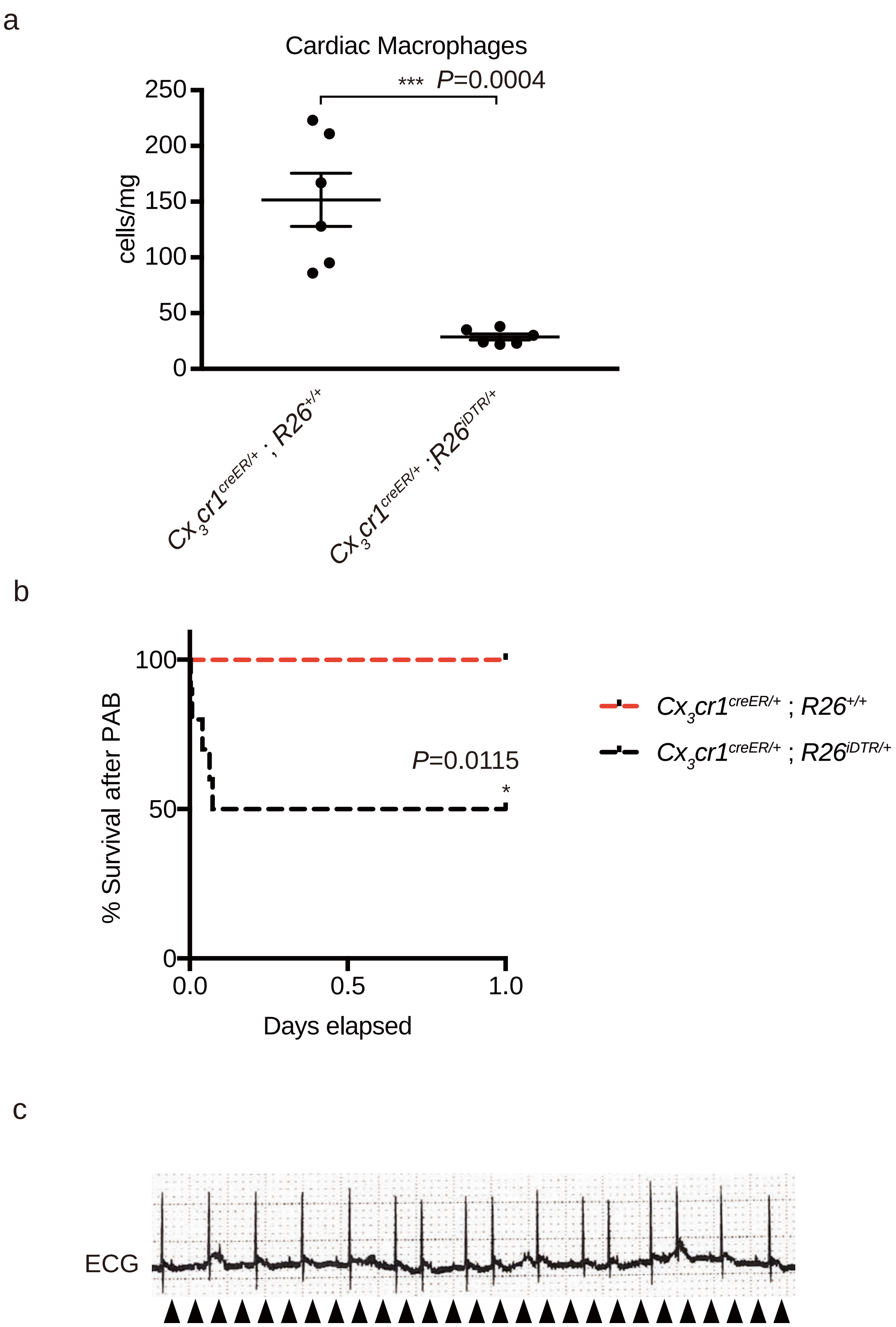
a
Cardiac Macrophages
*** P=0.0004
250
200
150
100
50
0
cells/mg
Cx3cr1creER/+ ; R26+/+
Cx3cr1creER/+ ;R26iDTR/+
b
100
50
0
% Survival after PAB
0.0
0.5
1.0
Days elapsed
P=0.0115
*
Cx3cr1creER/+ ; R26+/+
Cx3cr1creER/+ ; R26iDTR/+
c
ECG

Supplementary Figure 4. Functional and morphological analysis of *Areg*$^{-/-}$ hearts

a, b, Echocardiographic analysis of RV fractional shortening (FS) and wall thickness in WT and *Areg*$^{-/-}$ mice in the steady-state and 2 h after PAB. RV FS was determined in M mode with a parasternal long-axis view. No differences were found between WT and *Areg*$^{-/-}$ mice in the steady-state or after PAB. n=4 in each group. Two-way ANOVA followed by Tukey's multiple comparisons. Box plots show center line as median, box limits as upper and lower quartiles, whiskers as minimum to maximum values. c, Representative picrosirius red stained hearts from WT and *Areg*$^{-/-}$ mice 2 h after PAB. Note the absence of red staining in both ventricles. *n*=6 in each group. Bar, 1 mm.

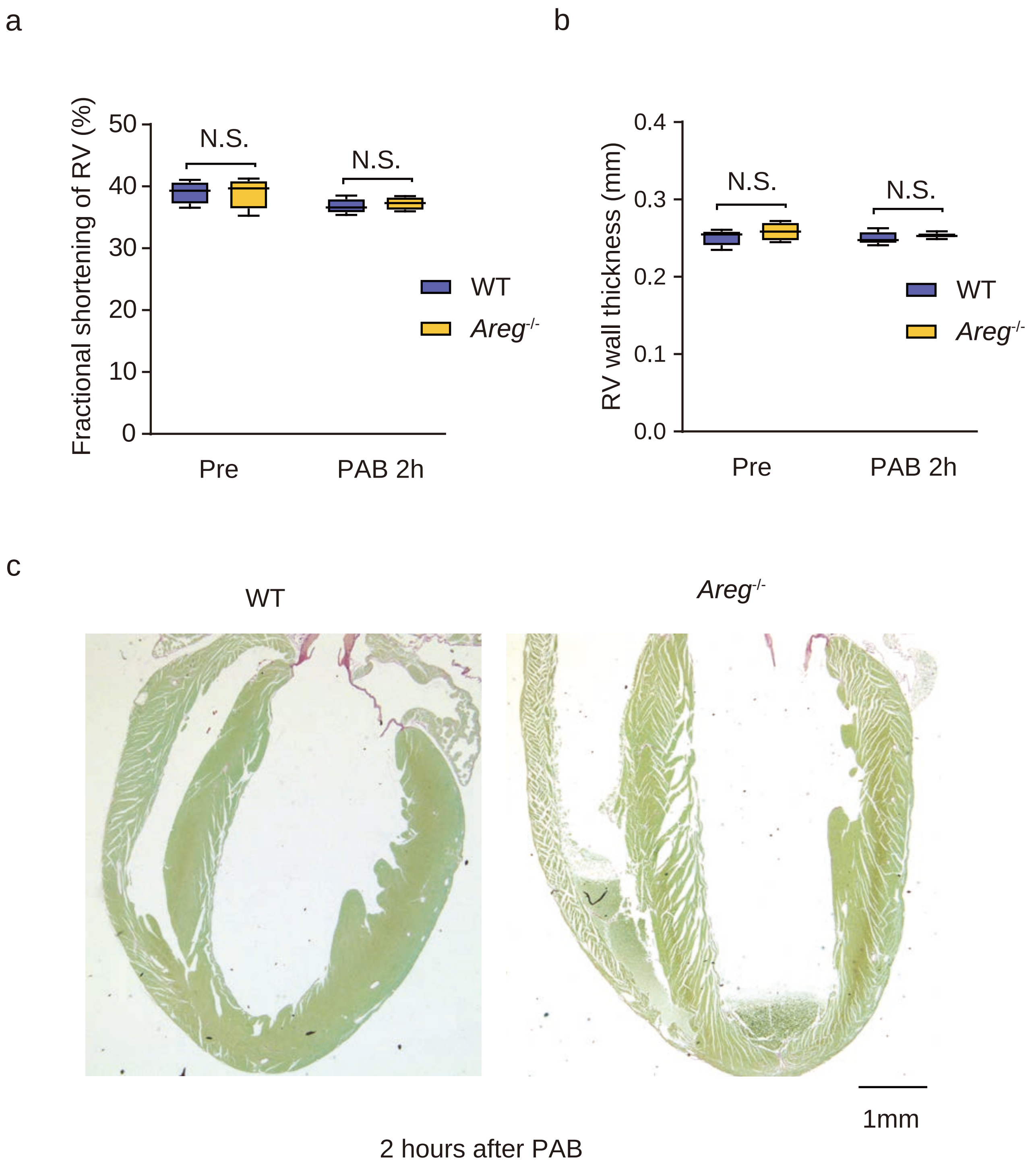
a
Fractional shortening of RV (%)
50
40
30
20
10
0
N.S.
N.S.
WT
Areg-/-
Pre
PAB 2h
b
RV wall thickness (mm)
0.4
0.3
0.2
0.1
0.0
N.S.
N.S.
WT
Areg-/-
Pre
PAB 2h
c
WT
Areg-/-
1mm
2 hours after PAB

Supplementary Figure 5. Effects of PAB on the hearts of macrophage-depleted *$CX_3CR1^{CreER/+}$;$R26^{iDTR/+}$* mice

a, Heart weight-to-body weight ratios measured in *$CX_3CR1^{CreER/+}$;$R26^{iDTR/+}$* mice after their sudden death following PAB. The control *$CX_3CR1^{CreER/+}$;$R26^{+/+}$* mouse was sacrificed when the *$CX_3CR1^{CreER/+}$;$R26^{iDTR/+}$* mouse died at all such times. n=5 in each group, two-tailed unpaired Student's *t*-test. Data are presented as mean values ± SEM. All analyzed *$CX_3CR1^{CreER/+}$;$R26^{iDTR/+}$* mice were suddenly died and control *$CX_3CR1^{CreER/+}$;$R26^{+/+}$* mice were sacrificed within 2 days after PAB. b, Histological analysis; shown are H.E. stained sections of representative hearts. Bar, 1 mm.

a

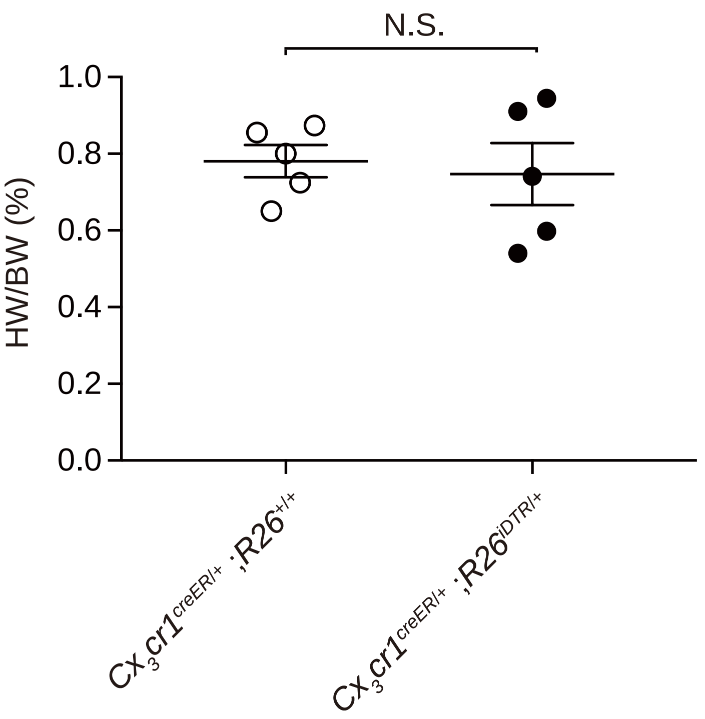
N.S.
1.0
0.8
0.6
0.4
0.2
0.0
HW/BW (%)
Cx3cr1creER/+;R26+/+
Cx3cr1creER/+;R26iDTR/+

b

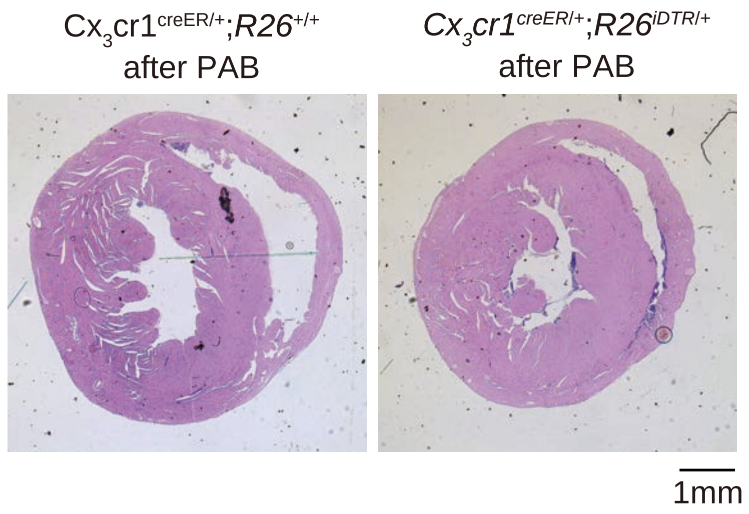
Cx3cr1creER/+;R26+/+
after PAB
Cx3cr1creER/+;R26iDTR/+
after PAB
1mm

Supplementary Figure 6. Single-cell RNA-seq of cardiac immune cells

A single-cell RNA-seq dataset (ArrayExpress E-MTAB-7376)[2] was integrated using Seurat ver. 3[3] as described previously[4]. Expression levels of *Areg* and lineage marker genes for macrophages (*Cd68*), T cells (*Cd3d*), and B cells (*Cd79a*) are shown in t-SNE plots. Note that most *Areg*-expressing cells are *Cd68*$^{+}$.

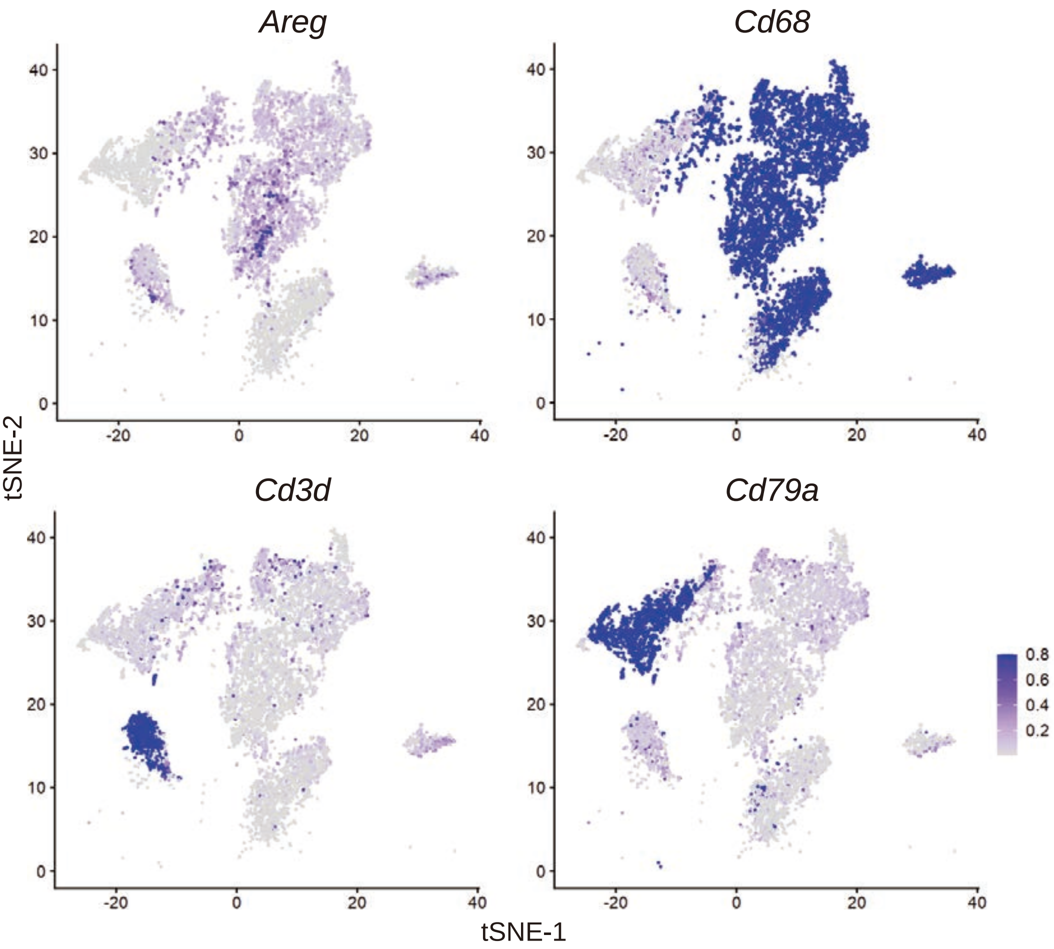

Areg
Cd68
Cd3d
Cd79a
tSNE-2
tSNE-1
0.8
0.6
0.4
0.2

Supplementary Figure 7. Wide QRS tachycardia in *Areg*$^{-/-}$ mice after isoproterenol

Representative wide QRS tachycardia recorded from an *Areg*$^{-/-}$ mouse administered isoproterenol infusion is shown on an expanded scale. Bar, 200 ms.

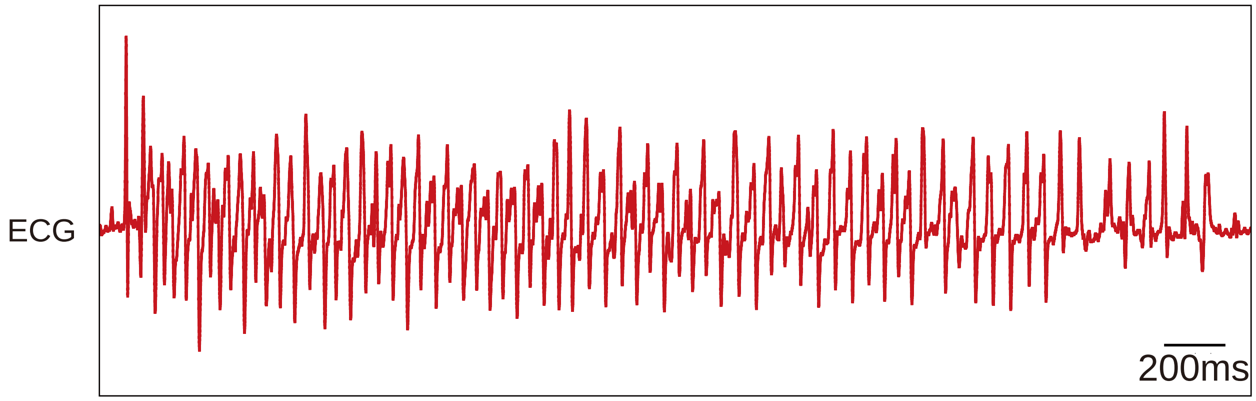
ECG
200ms

Supplementary Figure 8. Cx43 localization after PAB in WT and *Areg*$^{-/-}$ mice

Representative images showing immunohistochemical staining of Cx43 in the RVs of WT and *Areg*$^{-/-}$ mice 2 h after PAB. Bars, 100 μm.

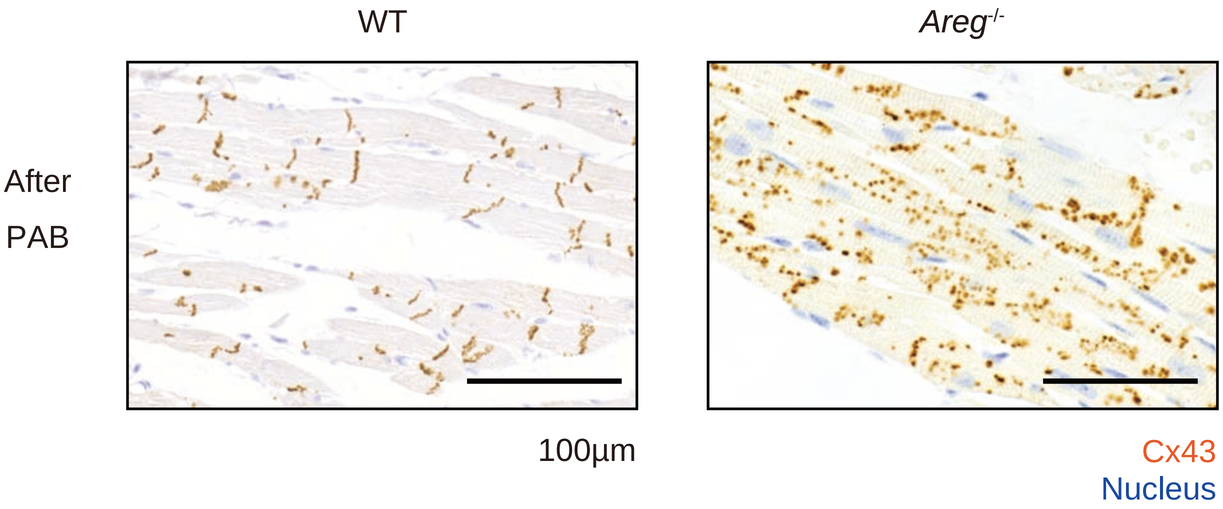
WT
Areg$^{-/-}$
After
PAB
100µm
Cx43
Nucleus

Supplementary Figure 9. Confocal microscopic imaging of Cx43 and N-cadherin

Shown are confocal micrographs of LVs in WT and *Areg*$^{-/-}$ mice. N-cadherin expression points to intercalated discs. The mislocation of Cx43 is also apparent in RV. White arrowheads indicate lateralization of Cx43 in *Areg*$^{-/-}$ hearts. Bar, 20 µm.

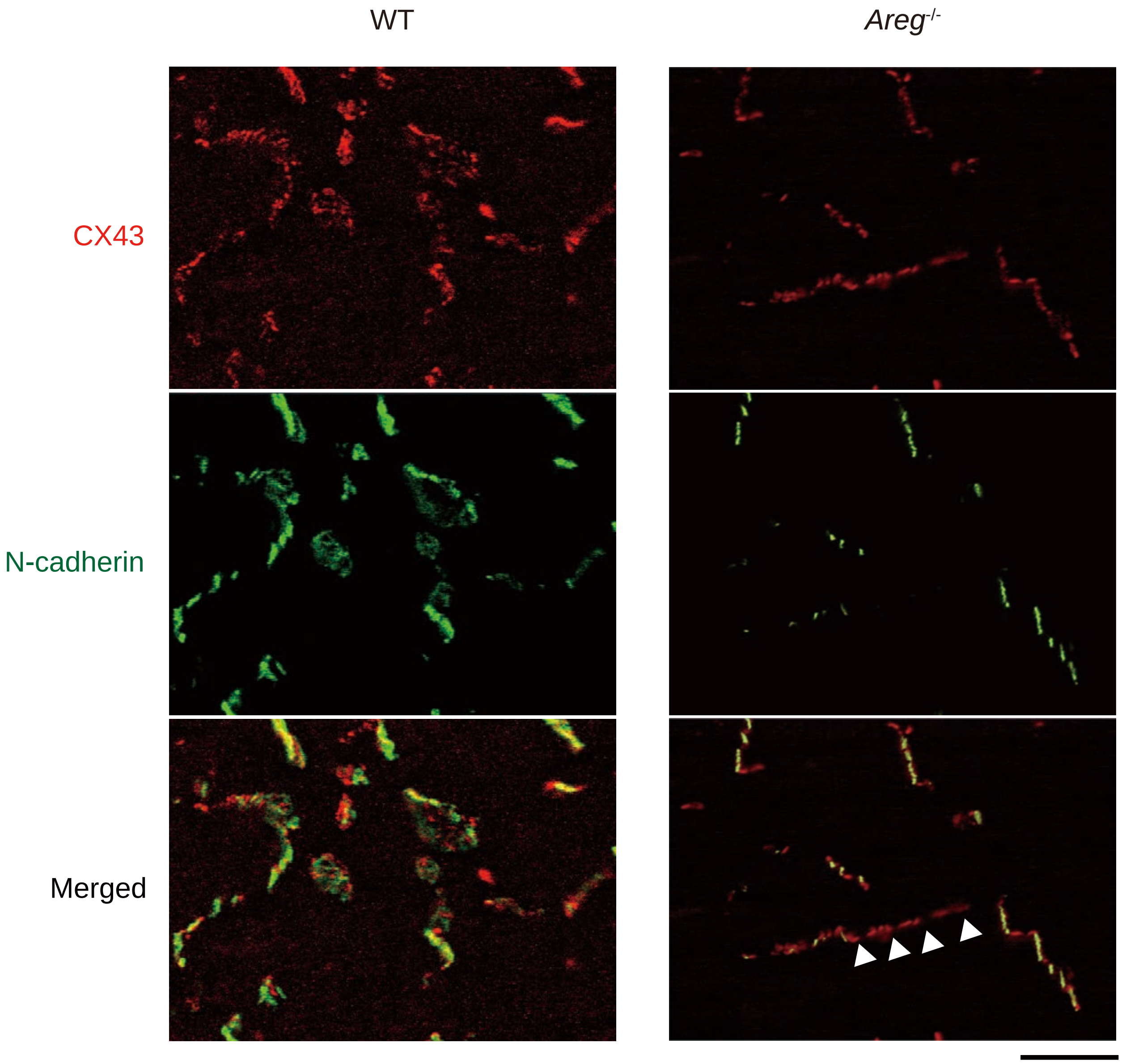

WT
Areg-/-
CX43
N-cadherin
Merged

Supplementary Figure 10. Hemichannel blocking rescued *Areg*$^{-/-}$ mice administered isoproterenol from sudden death

WT and *Areg*$^{-/-}$ mice were intraperitoneally administered a bolus of isoproterenol (5 mg/kg). The *Areg*$^{-/-}$ mice were also intraperitoneally administered Gap19, a selective Cx43 hemichannel blocker, or vehicle (PBS) 30 min before isoproterenol administration. Survival rates are shown. n=14 for WT mice, n=10 for *Areg*$^{-/-}$ mice with Gap19, and n=14 for *Areg*$^{-/-}$ mice without Gap19, *P<0.0001, #p<0.05 as indicated, N.S., not significant as indicated, Log-rank followed by post hoc Bonferroni's correction.

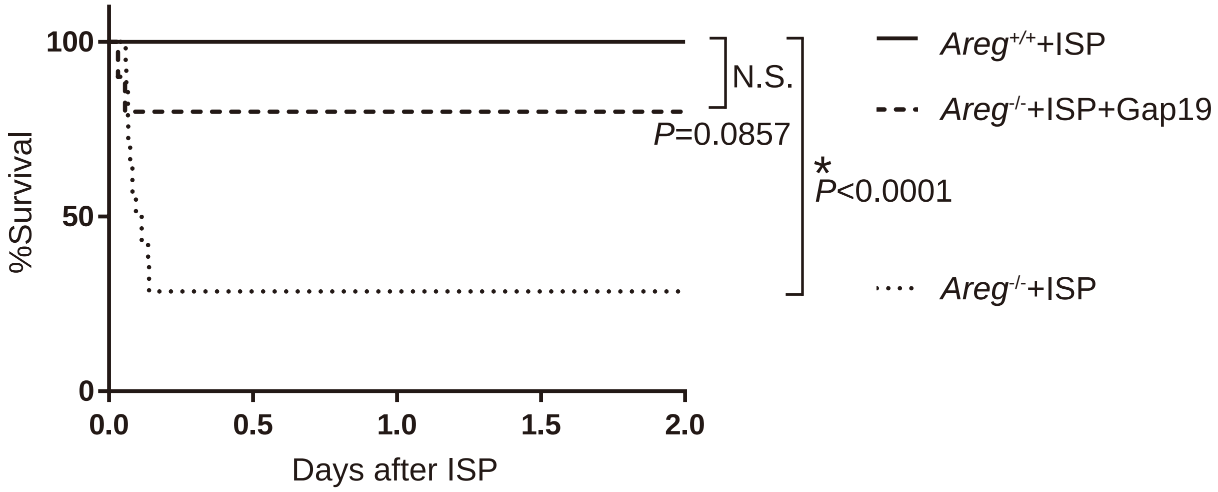

100
50
0
%Survival
0.0
0.5
1.0
1.5
2.0
Days after ISP
N.S.
P=0.0857
*
P<0.0001
Areg+/++ISP
Areg-/-+ISP+Gap19
Areg-/-+ISP

Supplementary Figure 11. The effects of EGFR ligands on conduction velocity

The conduction velocity of contraction propagation in cultured cardiomyocytes was measured using a SI8000 cell motion imager (Sony). a, Primary cardiomyocytes were cocultured with or without primary cardiac macrophages ($CD45^+CD11b^+F4/80^+Ly6G^-Ly6C^{lo}$). n=3 in each group, ****P*<0.001, two-tailed unpaired Student's *t*-test. Data are presented as mean values ± SEM. b, AREG was added to the culture medium, and conduction velocities were measured at the indicated times after its addition. The data at 0 h indicate the baseline prior to AREG addition. n=13, 12, 12 and 12, respectively, **P*<0.05 vs baseline. one-way ANOVA followed by Holm-Sidak multiple comparion test. Data are presented as mean values ± SEM. c, HBEGF was added to the cardiomyocyte cultures, and the conduction velocities were measured at the time of 0 and 10 h, n=12 in each group, two-tailed unpaired Student's *t*-test. Data are presented as mean values ± SEM.

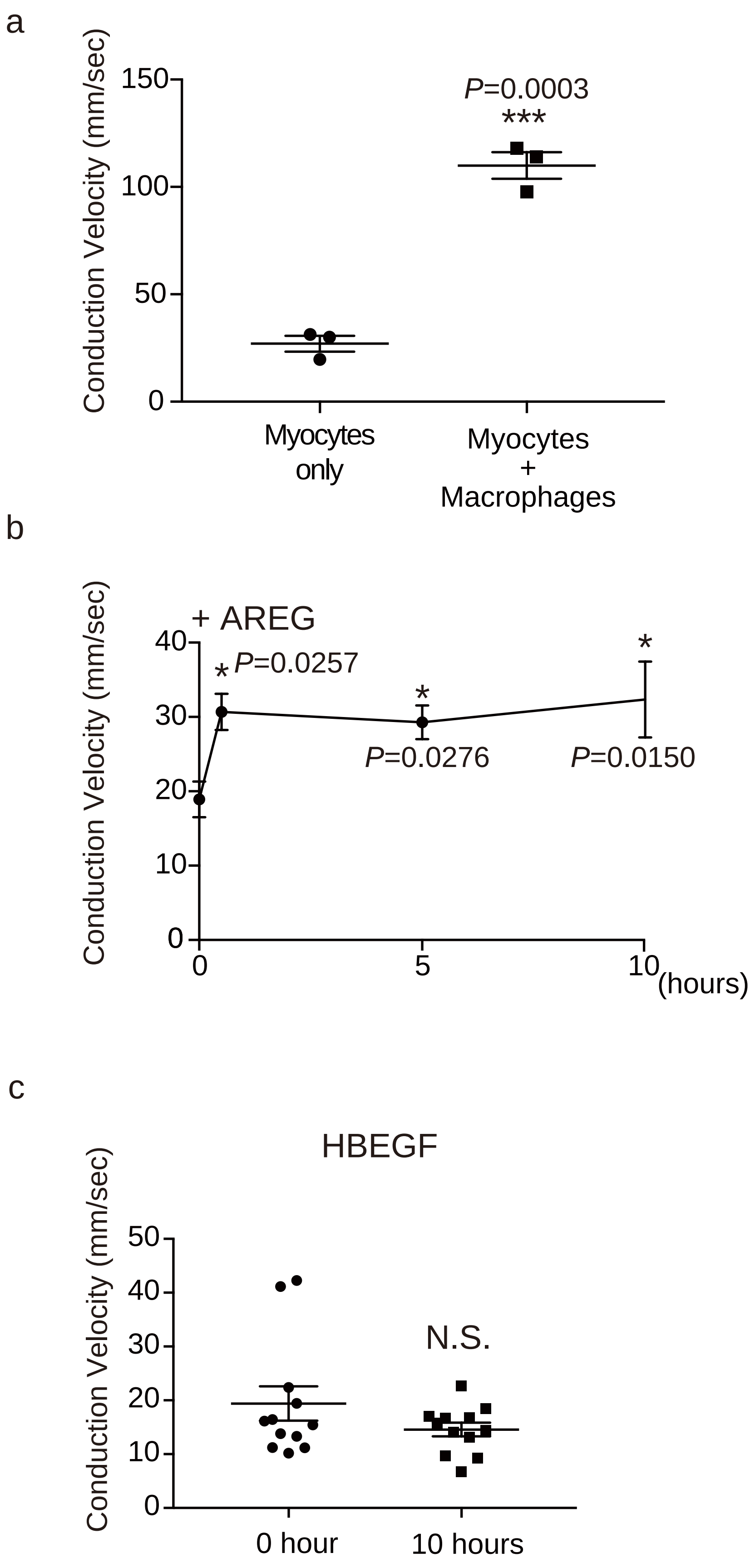
a
Conduction Velocity (mm/sec)
150
100
50
0
P=0.0003
***
Myocytes only
Myocytes + Macrophages
b
Conduction Velocity (mm/sec)
+ AREG
40
30
20
10
0
* P=0.0257
*
*
P=0.0276
P=0.0150
0
5
10
(hours)
c
HBEGF
Conduction Velocity (mm/sec)
50
40
30
20
10
0
N.S.
0 hour
10 hours

Supplementary Figure 12. Optical mapping of electrical activation in the heart

Conduction properties in hearts in WT and *Areg*$^{-/-}$ mice were evaluated by optical mapping. a, Representative isochrone maps of activation during constant stimulation [basic cycle length (BCL) = 150 ms] of WT and *Areg*$^{-/-}$ mice. b-c, Conduction velocities in the longitudinal (CV_l) (b) and transverse (CV_t) (c) directions, and the anisotropic ratio (CV_l/CV_t). n=4 for WT mice, n=6 for *Areg*$^{-/-}$ mice, $*P<0.05$, two-tailed unpaired Student's *t*-test. Data are presented as mean values ± SEM.

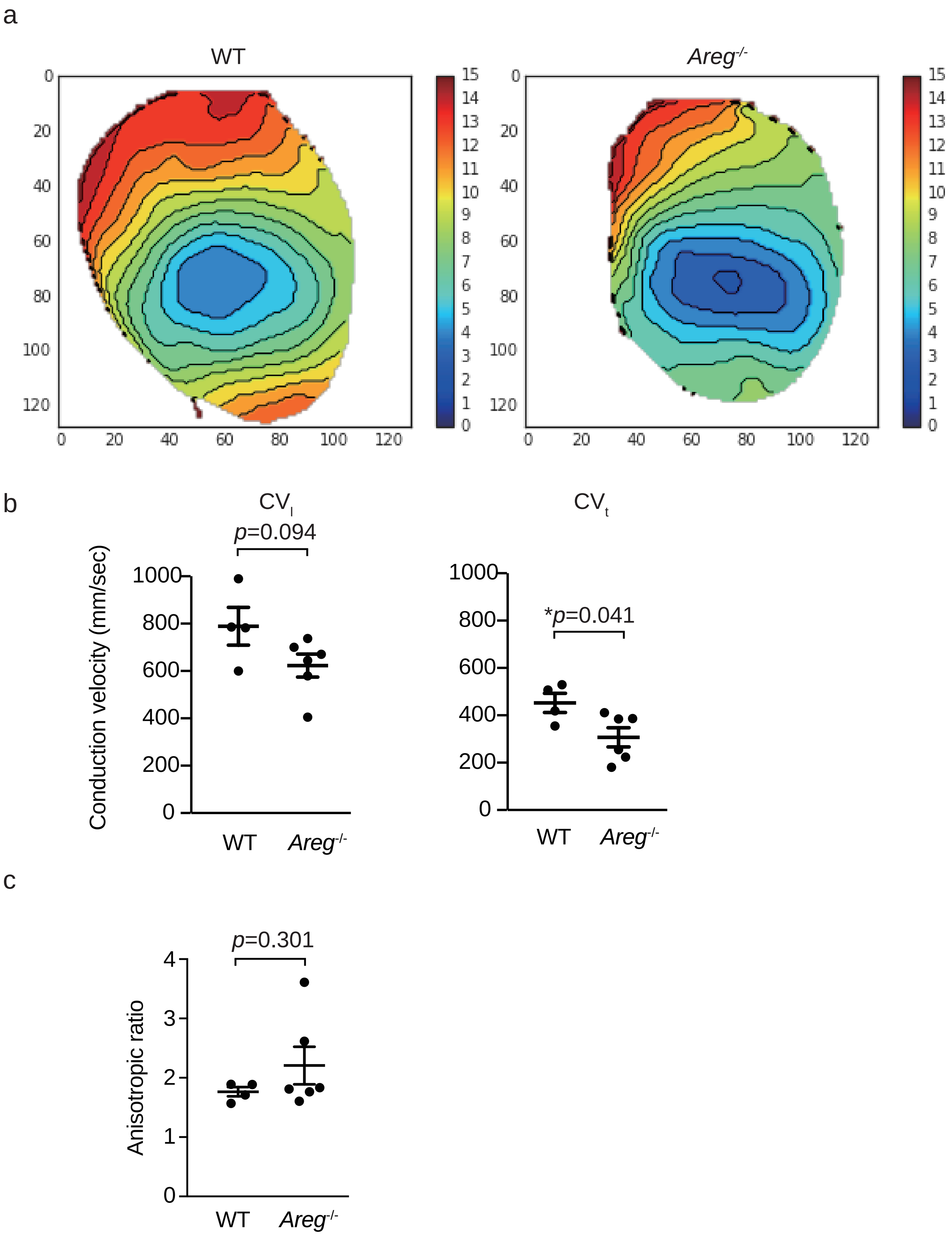
a
WT
Areg-/-
0
20
40
60
80
100
120
15
14
13
12
11
10
9
8
7
6
5
4
3
2
1
0
b
CVl
p=0.094
CVt
*p=0.041
Conduction velocity (mm/sec)
1000
800
600
400
200
0
WT
Areg-/-
c
p=0.301
Anisotropic ratio
4
3
2
1
0
WT
Areg-/-

Supplementary Figure 13. AREG-treated cells exhibit greater gap junction formation than untreated cells expressing the same levels of Cx43 protein

Cx43-GFP plasmid was transfected into HeLa cells, and the cells were treated for 5 h with or without AREG (100 ng/mL), as indicated. Cx43 can be visualized as green signals. Representative images of Cx43 gap junction plaques (white arrows) at the cell-cell borders are shown. Cell borders are marked by dashed lines in the upper panels. Bar, 10 μm.

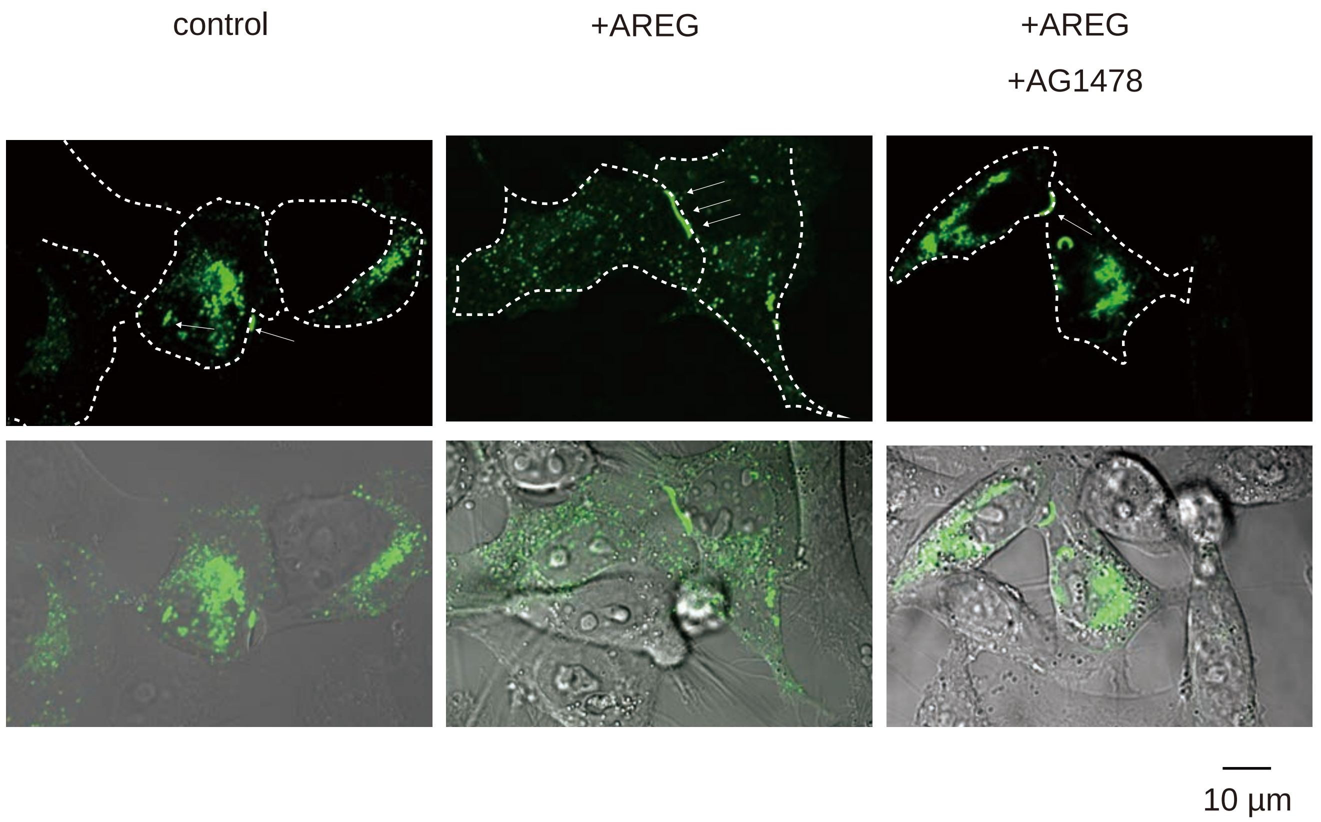
control
+AREG
+AREG
+AG1478
10 µm

Supplementary Figure 14. Analysis of serine residues affected by AREG-induced phosphorylation of Cx43

a, HeLa cells were transfected with expression vectors encoding GFP-tagged Cx43 or mutant Cx43 in which serine residues that could be phosphorylated via known pathways were replaced with alanine, and then incubated with AREG (100 ng/ml) for 30 min. The cells were harvested and the cell lysates were subjected to Phos-tag SDS-PAGE and immunoblotted with anti-GFP antibody. The phosphorylation pathways and corresponding serine residues were: Akt, S373; PKC, S368; cAMP, S365; CK1, S325/328/330; and MAPK, S255/262/279/282. Cx43 phosphorylation was reduced by S365A, S325/328/330/A, or S255/262/279/282A mutations. The total amount of Cx43 in each sample was shown by western blotting of λ-phosphatase-treated same samples. The most phosphorylated Cx43 band intensity was normalized by the total Cx43 band intensity of the same sample, one-way ANOVA, $p<0.0001$; Tukey's post hoc test *p=0.0131, †p =0.0010, and § p <0.0001 vs. Cx43(WT)-GFP, n=4 in each group. Data are presented as mean values ± SEM. b, HeLa cells were transfected with a vector encoding GFP-tagged wild-type Cx43 and treated with HBEGF (10 ng/ml) or TGF-α (100 ng/ml) , AREG (100 ng/ml) for 30 min. Note that TGF-α promoted patterns of Cx43 phosphorylation that differed from those promoted by AREG.

a

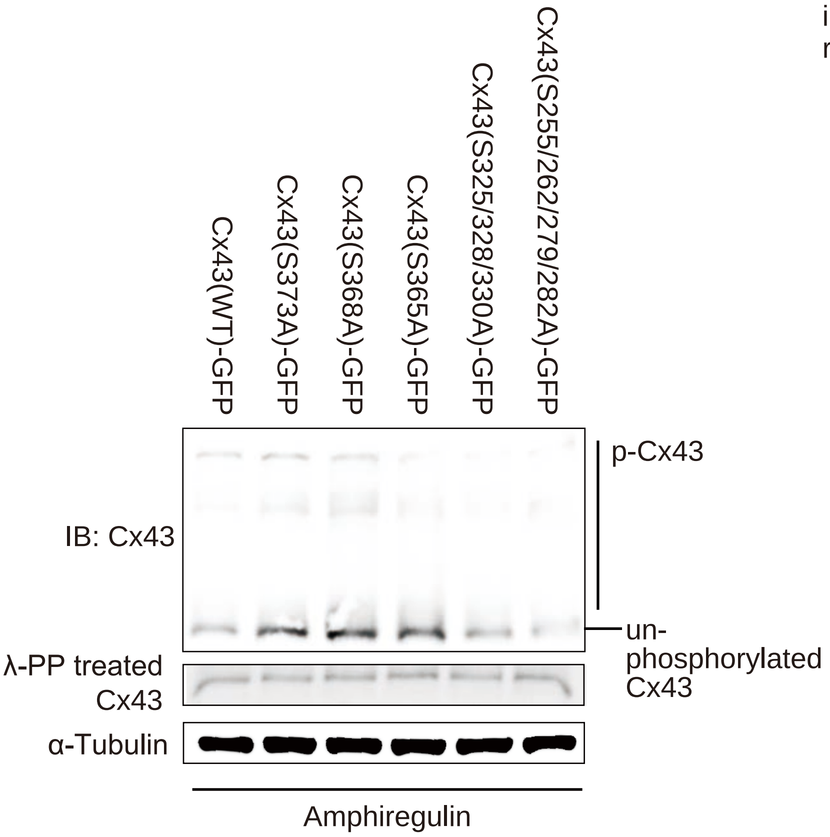
Cx43(WT)-GFP
Cx43(S373A)-GFP
Cx43(S368A)-GFP
Cx43(S365A)-GFP
Cx43(S325/328/330A)-GFP
Cx43(S255/262/279/282A)-GFP
IB: Cx43
p-Cx43
un-phosphorylated Cx43
λ-PP treated Cx43
α-Tubulin
Amphiregulin

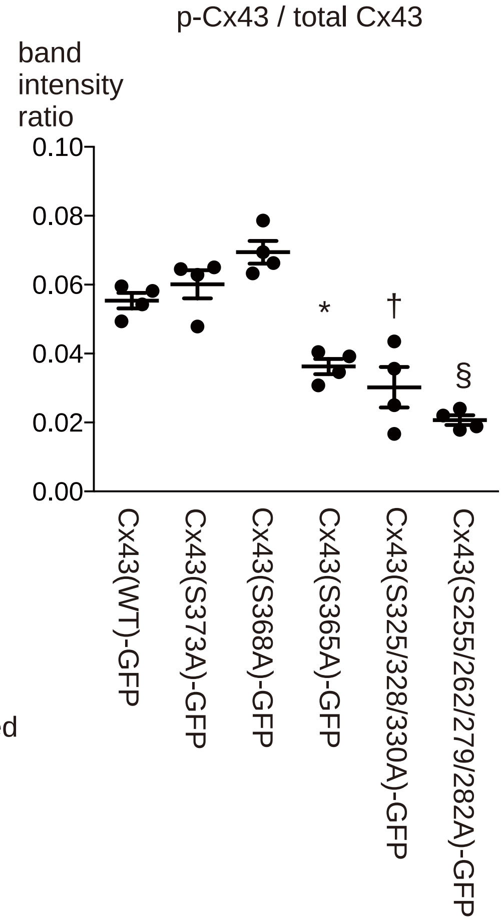
p-Cx43 / total Cx43
band intensity ratio
0.10
0.08
0.06
0.04
0.02
0.00
*
†
§
Cx43(WT)-GFP
Cx43(S373A)-GFP
Cx43(S368A)-GFP
Cx43(S365A)-GFP
Cx43(S325/328/330A)-GFP
Cx43(S255/262/279/282A)-GFP

b

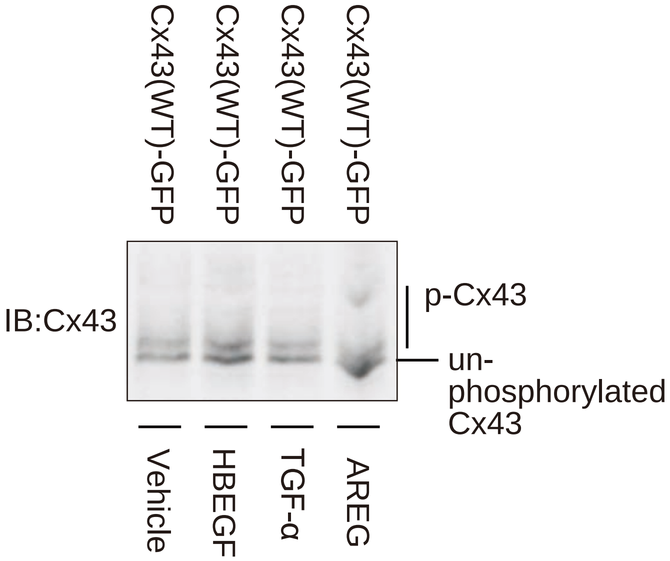
Cx43(WT)-GFP
Cx43(WT)-GFP
Cx43(WT)-GFP
Cx43(WT)-GFP
IB:Cx43
p-Cx43
un-phosphorylated Cx43
Vehicle
HBEGF
TGF-α
AREG

Supplementary Figure 15. mRNA expression of EGFR ligands in cardiac-resident macrophages

qPCR analysis of *Areg* and *Hbegf* expression in cardiac-resident macrophages from WT and *Areg*$^{-/-}$ mice in the steady state. N.D.: not detected. mRNA levels were first normalized to those of 18s rRNA and then further normalized to the level of cardiac-resident macrophages of WT mice. n=4 in each group. *P<0.01, two-tailed unpaired Student's *t*-test. Box plots show center line as median, box limits as upper and lower quartiles, whiskers as minimum to maximum values.

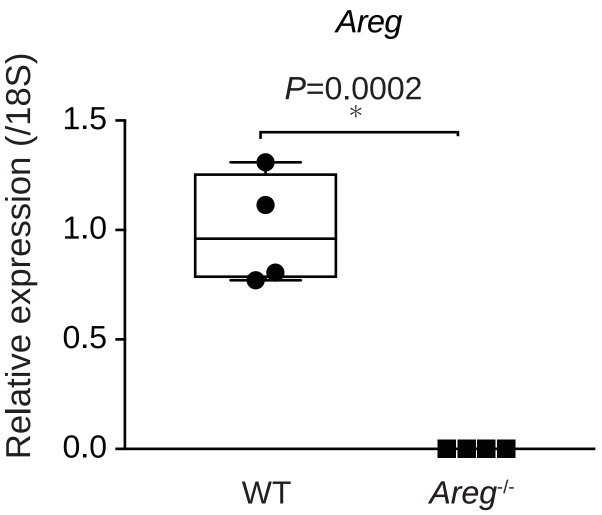
Areg
P=0.0002
*
Relative expression (/18S)
1.5
1.0
0.5
0.0
WT
Areg-/-

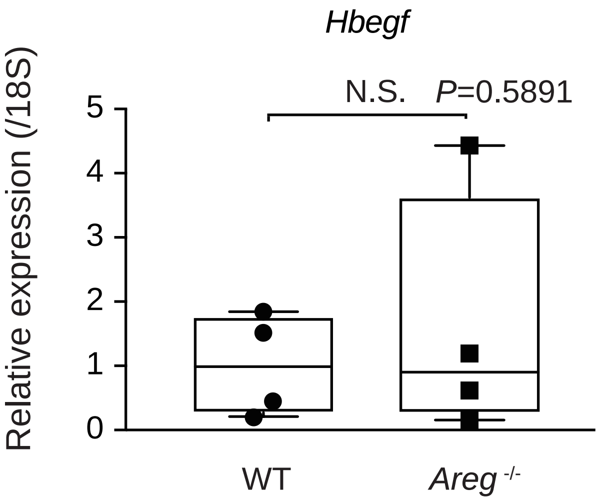
Hbegf
N.S. P=0.5891
Relative expression (/18S)
5
4
3
2
1
0
WT
Areg -/-

Supplementary Figure 16. FACS strategy

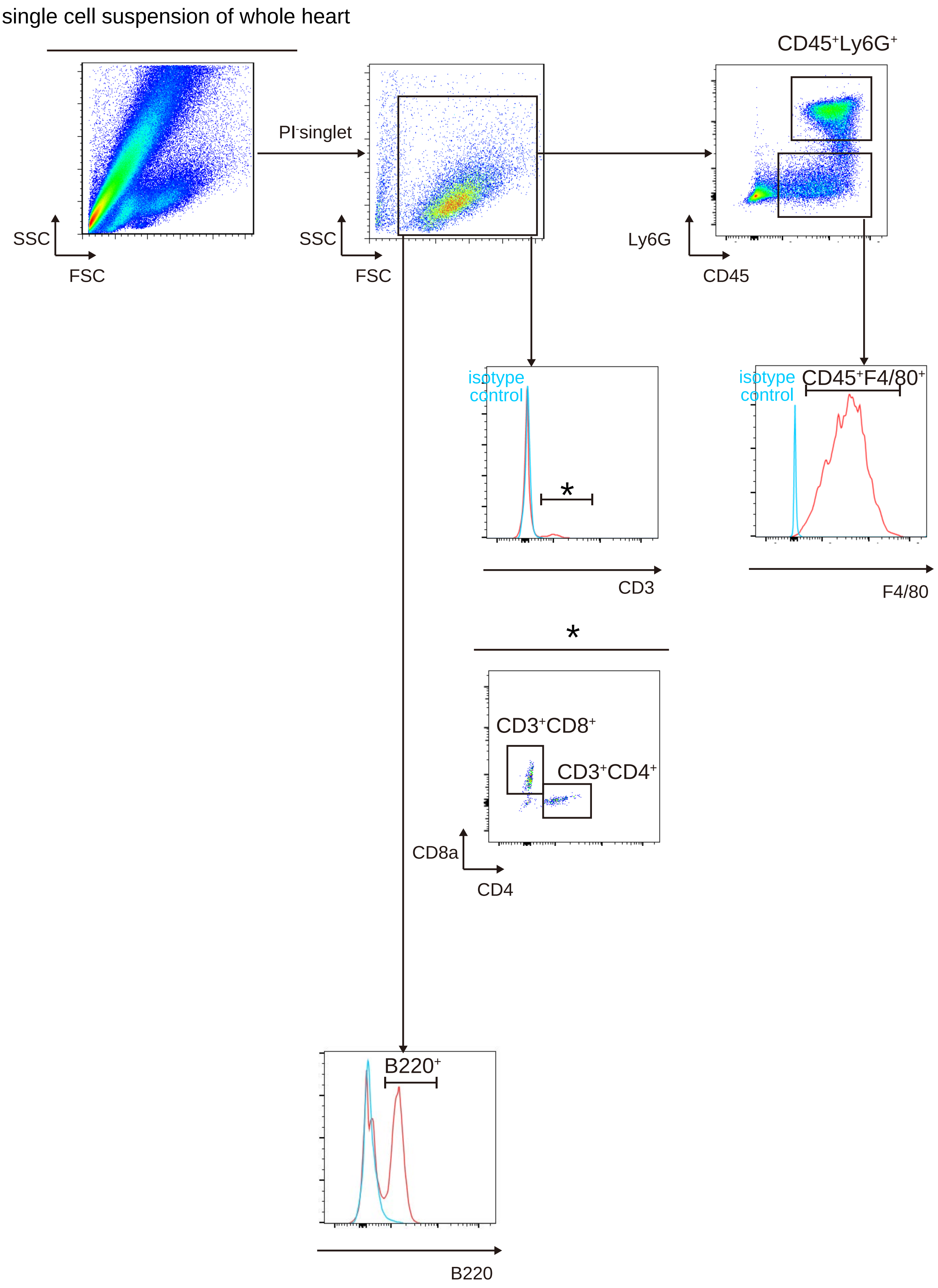

single cell suspension of whole heart
PI-singlet
CD45+Ly6G+
SSC
FSC
SSC
FSC
Ly6G
CD45
isotype control
*
CD3
isotype control
CD45+F4/80+
F4/80
*
CD3+CD8+
CD3+CD4+
CD8a
CD4
B220+
B220

Supplementary Figure 17. Uncropped western blotting images

Fig.3a

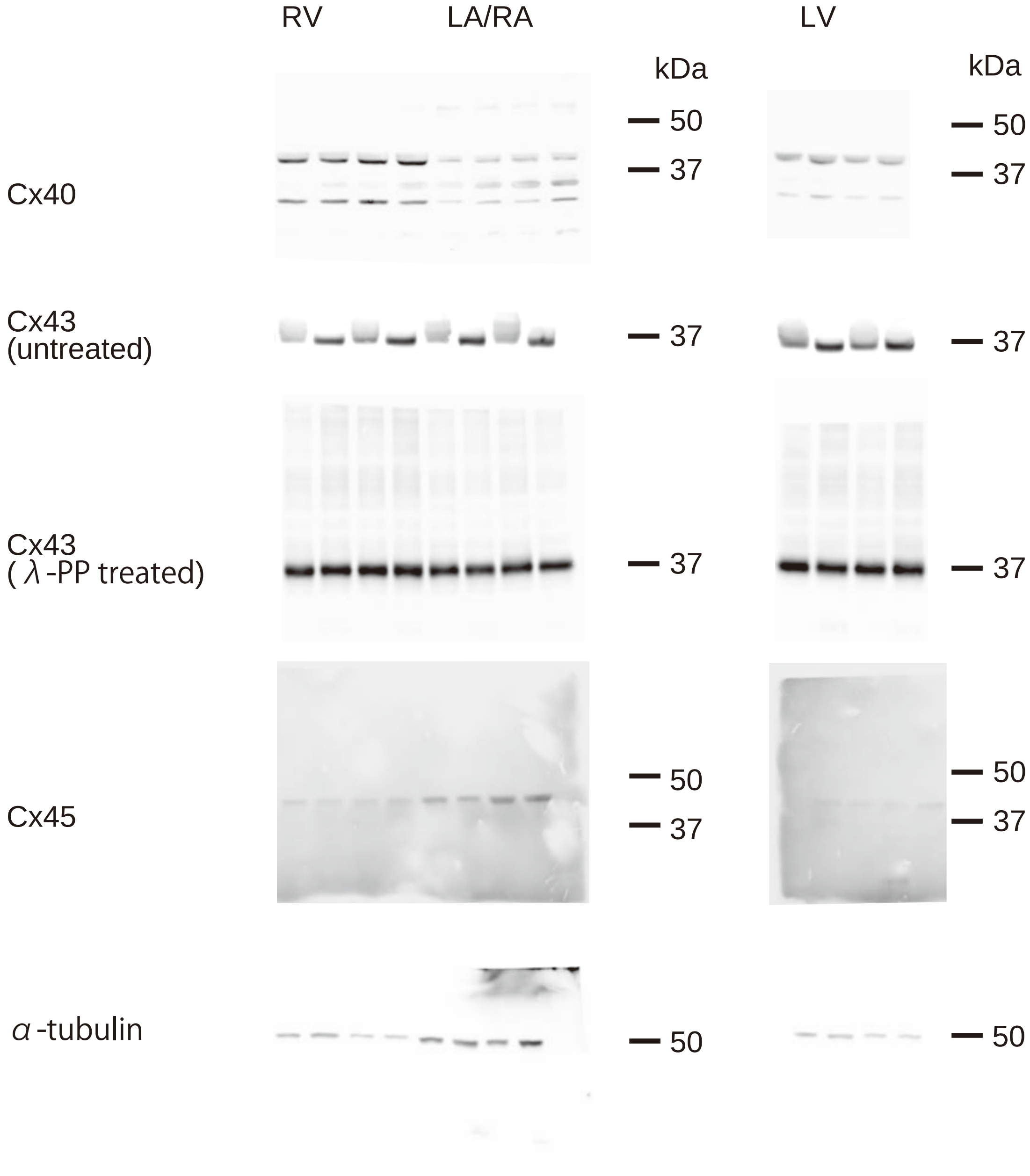
RV
LA/RA
LV
kDa
kDa
50
37
Cx40
Cx43
(untreated)
37
Cx43
(λ -PP treated)
37
Cx45
50
37
α -tubulin
50

Fig.4g

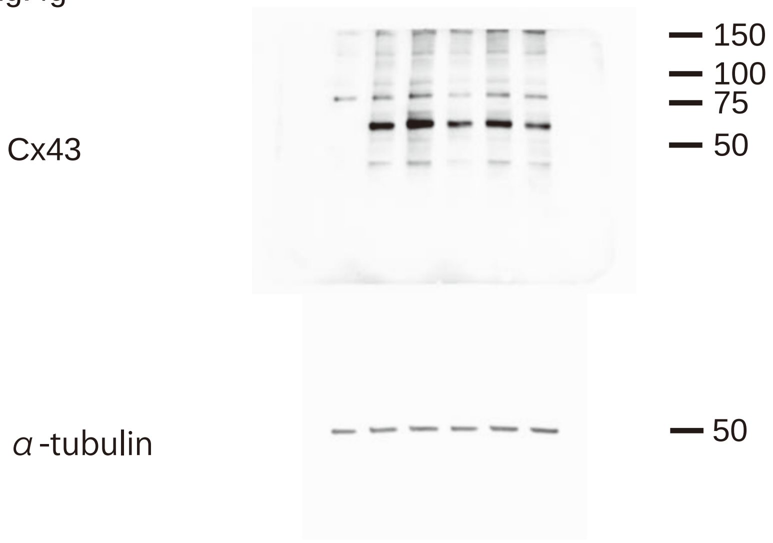
150
100
75
50
Cx43
α -tubulin
50

Supplementary Figure 18. Uncropped western blotting images

Uncropped western blotting images

Supplementary Fig. 14a

Cx43

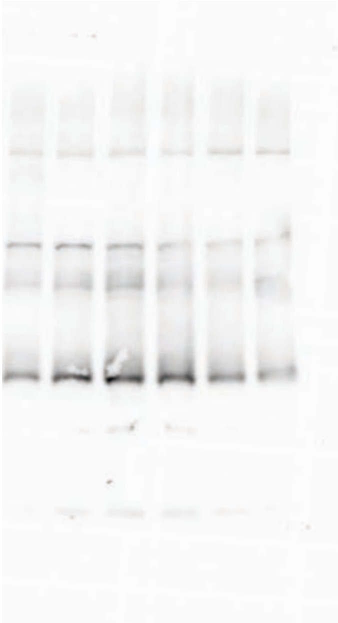

α-Tubulin

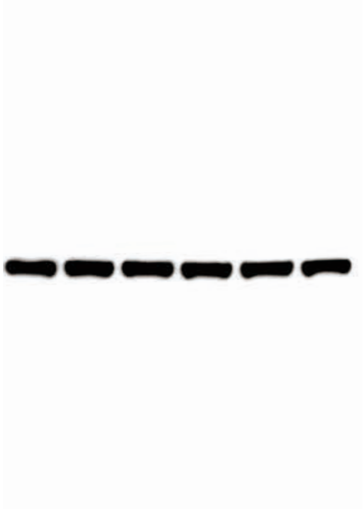

kDa

50

λ-PP treated Cx43

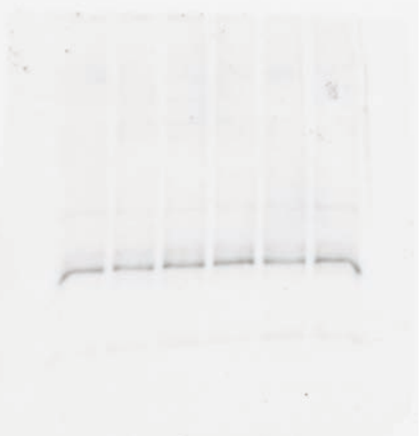

Supplementary Fig. 14b

Cx43

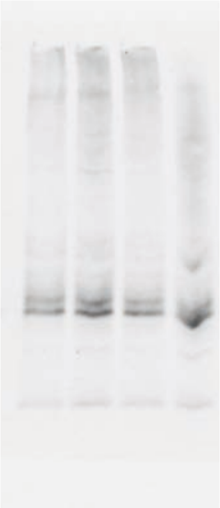

Supplement: Supplementary file 1 — Supplementary Information [file 41467_2021_22178_MOESM1_ESM.pdf]
