# Supplementary material for: Cardiac macrophages prevent sudden death during heart stress
Source: Nat Commun. 2021 Mar 26;12:1910. doi: 10.1038/s41467-021-22178-0 (PMC7997915; doi:10.1038/s41467-021-22178-0)
Supplement: Supplementary file 2 — Reporting Summary [file 41467_2021_22178_MOESM2_ESM.pdf]

nature research

Corresponding author(s): Katsuhito Fujiu
Ichiro Manabe

Last updated by author(s): Feb 7, 2021

Reporting Summary

Nature Research wishes to improve the reproducibility of the work that we publish. This form provides structure for consistency and transparency in reporting. For further information on Nature Research policies, see our Editorial Policies and the Editorial Policy Checklist.

Statistics

For all statistical analyses, confirm that the following items are present in the figure legend, table legend, main text, or Methods section.

n/a	Confirmed	
☐	☒	The exact sample size (*n*) for each experimental group/condition, given as a discrete number and unit of measurement
☐	☒	A statement on whether measurements were taken from distinct samples or whether the same sample was measured repeatedly
☐	☒	The statistical test(s) used AND whether they are one- or two-sided<br>*Only common tests should be described solely by name; describe more complex techniques in the Methods section.*
☒	☐	A description of all covariates tested
☐	☒	A description of any assumptions or corrections, such as tests of normality and adjustment for multiple comparisons
☐	☒	A full description of the statistical parameters including central tendency (e.g. means) or other basic estimates (e.g. regression coefficient) AND variation (e.g. standard deviation) or associated estimates of uncertainty (e.g. confidence intervals)
☐	☒	For null hypothesis testing, the test statistic (e.g. *F*, *t*, *r*) with confidence intervals, effect sizes, degrees of freedom and *P* value noted<br>*Give P values as exact values whenever suitable.*
☒	☐	For Bayesian analysis, information on the choice of priors and Markov chain Monte Carlo settings
☐	☒	For hierarchical and complex designs, identification of the appropriate level for tests and full reporting of outcomes
☒	☐	Estimates of effect sizes (e.g. Cohen's *d*, Pearson's *r*), indicating how they were calculated

Our web collection on statistics for biologists contains articles on many of the points above.

Software and code

Policy information about availability of computer code

Data collection	BD FACSDIVA v8.0.2, ImageJ 1.53a, LabChart pro ver. 8
Data analysis	GraphPad Prism 8, Microsoft Excel 2019, FlowJo 10.7.1, Seurat ver. 3

For manuscripts utilizing custom algorithms or software that are central to the research but not yet described in published literature, software must be made available to editors and reviewers. We strongly encourage code deposition in a community repository (e.g. GitHub). See the Nature Research guidelines for submitting code & software for further information.

Data

Policy information about availability of data

All manuscripts must include a data availability statement. This statement should provide the following information, where applicable:

- Accession codes, unique identifiers, or web links for publicly available datasets
- A list of figures that have associated raw data
- A description of any restrictions on data availability

Raw data for single cell RNA-seq dataset of cardiac immune cells are available from Array Express under accession code E-MTAB-7376. The authors declare that the data supporting the findings of this study are available within the article and its Supplementary Information Files or from the corresponding authors upon reasonable request.

Field-specific reporting

Please select the one below that is the best fit for your research. If you are not sure, read the appropriate sections before making your selection.

- [x] Life sciences
- [ ] Behavioural & social sciences
- [ ] Ecological, evolutionary & environmental sciences

For a reference copy of the document with all sections, see nature.com/documents/nr-reporting-summary-flat.pdf

Life sciences study design

All studies must disclose on these points even when the disclosure is negative.

Sample size	Sample size was determined during experimental design based on the type of measurements made and our extensive experience with data variation for each technique. A power calculation was not done. Animal sample size estimates were determined using power calculations. GraphPad PRISM software was used for statistical analyses. Unpaired t test were used to determine the power (a=0.05, two-tailed). We observed many statistically significant effects in the data, indicating that the effective sample size was sufficient for studying the phenomena of interest.
Data exclusions	No data was excluded.
Replication	Each experiment was repeated three times with a minimum of n = 4 biological replicates, unless otherwise stated. Only reproducible experiments are reported in this manuscript.
Randomization	Samples were randomly assigned.
Blinding	Investigators were not blinded to group allocation because treatments and data collection were performed by the same people. The analyses, including measurement of echocardiography and pathological analyses, and the preparation of cells for flow cytometric analysis, were performed by another independent investigator.

Reporting for specific materials, systems and methods

We require information from authors about some types of materials, experimental systems and methods used in many studies. Here, indicate whether each material, system or method listed is relevant to your study. If you are not sure if a list item applies to your research, read the appropriate section before selecting a response.

Materials & experimental systems

n/a	Involved in the study	
[ ]	[x]	Antibodies
[ ]	[x]	Eukaryotic cell lines
[x]	[ ]	Palaeontology and archaeology
[ ]	[x]	Animals and other organisms
[x]	[ ]	Human research participants
[x]	[ ]	Clinical data
[x]	[ ]	Dual use research of concern

Methods

n/a	Involved in the study	
[x]	[ ]	ChIP-seq
[ ]	[x]	Flow cytometry
[x]	[ ]	MRI-based neuroimaging

Antibodies

Antibodies used

FACS antibodies:
Anti-CD45.2-V500(562129, BD bioscience, 1:100)
Anti-CD45.2-APC(109814, BioLegend, 1:100)
Anti-CD4-PerCP/Cy5.5(100433, BioLegend, 1:100)
Anti-CD8a-PerCP/Cy5.5(100733, BioLegend, 1:100)
Anti-CD11b-PerCP/Cy5.5(45-0112-80, eBioscience, 1:100)
Anti-Ly6g-PerCP/Cy5.5(127615, BioLegend, 1:100)
Anti-B220-PerCP/Cy5.5(103235, BioLegend, 1:100)
Anti-CD11b-BV421(101251, BioLegend, 1:100)
Anti-CD64-APC(139306, BioLegend, 1:100)
Anti-F4/80-PE(123110, BioLegend, 1:100)
Anti-Ly6c-PE-Cy7(128018, BioLegend, 1:100)
Anti-HBEGF(sc-365182, Santa Cruz Biotechnology, 1:100)
Anti-amphiregulin(AF989, R&D systems, 1:100)
Donkey anti-Goat IgG (H+L) Cross-Adsorbed Secondary Antibody, Alexa Fluor 488 (A11055, Invitrogen, 1:300)
Goat anti-Mouse IgG (H+L) Cross-Adsorbed Secondary Antibody, Alexa Fluor 488 (A11001, Invitrogen, 1:300)

In vivo administration antibodies:
Anti-Ly6G (127632, BioLegend)

Western blotting antibodies:
Anti-Cx40(AB1726, Merck Millipore, 1:500)
Anti-Cx43(C6219, Sigma, 1:2000)
Anti-Cx45(AB1745, Merck Millipore, 1:2000)
Anti-α-tubulin(T6199, Sigma, 1:1000)
Anti-rabbit IgG-HRP(7074, Cell Signaling Technology, 1:5000)
Anti-mouse IgG-HRP(7076, Cell Signaling Technology, 1:5000)

Immunohistochemistry antibodies:
Anti-F4/80(MCA497G, Serotec,1:400)
Anti-Ly6G(127601, BioLegend, 1:100)
Anti-B220(CD45R)(550286, BD bioscience, 1:100)
Anti-CD3(GTX42110, GeneTex, 1:100)
Anti-Cx43(C6219, Sigma, 1:2000)
Anti-N-cadherin(33-3900, ThermoFisher, 1:500)
Alexa-Fluor-488-conjugated anti-mouse-IgG(A-11001, ThermoFisher, 1:2000)
Alexa-Fluor-635-conjugated anti-rabbit-IgG(A-31576, ThermoFisher, 1:2000)

Validation

All the antibodies were validated by manufacturer, and the information about the validation can be found on the manufacturer's website through the links below: https://www.bdbiosciences.com/en-us; https://www.biolegend.com; https://www.thermofisher.com/us/en/home.html; https://www.scbt.com/home; https://www.rndsystems.com; http://www.emdmillipore.com/US/en; https://www.sigmaaldrich.com/united-states.html; https://www.cellsignal.com/?country=USA; https://www.bio-rad.com; https://www.genetex.com.

Eukaryotic cell lines

Policy information about cell lines

Cell line source(s)	Human cervical cancer (HeLa) cells were obtained from the American Culture Collection(ATCC).
Authentication	The cell line is authenticated by STR method.
Mycoplasma contamination	The cell line was tested for mycoplasma contamination.
Commonly misidentified lines (See ICLAC register)	None of the used cell lines is listed in ICLAC database.

Animals and other organisms

Policy information about studies involving animals; ARRIVE guidelines recommended for reporting animal research

Laboratory animals	Male C57BL/6J mice were purchased from CLEA Japan, Inc. (Tokyo, Japan). Male Areg homozygous null (Areg-/-) mice were backcrossed to C57BL6/J mice over ten generations. Rag2-/-, Cd4-/-, and Cd8a-/- mice were purchased from Taconic (Germantown, NY). The 6-10 week old male mice were used in every assay. All mice were housed in separate cages in a pathogen-free environment at the University of Tokyo animal facility. Mice received standard mouse chow and water ad libitum.
Wild animals	This study did not involve wild animals.
Field-collected samples	This study did not involve field-collected samples.
Ethics oversight	All experiments were approved by the University of Tokyo Ethics Committee for Animal Experiments and strictly adhered to the guidelines for animal experiments at the University of Tokyo.

Note that full information on the approval of the study protocol must also be provided in the manuscript.

Flow Cytometry

Plots

Confirm that:

- [x] The axis labels state the marker and fluorochrome used (e.g. CD4-FITC).
- [x] The axis scales are clearly visible. Include numbers along axes only for bottom left plot of group (a 'group' is an analysis of identical markers).
- [x] All plots are contour plots with outliers or pseudocolor plots.
- [x] A numerical value for number of cells or percentage (with statistics) is provided.

Methodology

Sample preparation	For sorting of cardiac macrophages, cardiac tissues were homogenized as followed. Mice were anesthetized by intraperitoneal injection of a mixture of xylazine (10 mg/kg) and ketamine (100 mg/kg). The heart was exposed and perfused with 10 ml PBS from the left ventricle. Thereafter, the whole heart tissue was excised and mechanically minced using curved scissors. The tissue from 1 heart was then incubated in 1 ml of DMEM containing 450 U/ml of collagenase I (Sigma-Aldrich), 60 U/ml of hyaluronidase (Sigma-Aldrich), and 60 U/ml of DNase-I (Sigma-Aldrich) for 45 min at 37 °C. The cells in the suspension were vortexed for 20 sec, filtered through a 40 µm cell strainer, and washed by 12 ml cold HBSS supplemented with 2% FBS and 0.2% BSA. The cells were then centrifuged at 400 g for 5 min, washed with PBS, and resuspended in FACS buffer.
Instrument	FACSAria IIIa (Becton Dickinson)
Software	Data were collected using BD FACSDiva software v8.0.1 and analyzed using FlowJo software (Tree Star) v10.7.1.
Cell population abundance	We had not confirmed the purity of sorted cells by repeated flow cytometric analysis, but sorted cells by high-purity mode.
Gating strategy	Relevant gating strategies are shown in Supplementary Figure 16.

☒ Tick this box to confirm that a figure exemplifying the gating strategy is provided in the Supplementary Information.
